# Supplementary material for: Tuning network topology and vibrational mode localization to achieve ultralow thermal conductivity in amorphous chalcogenides
Source: Nat Commun. 2021 May 14;12:2817. doi: 10.1038/s41467-021-22999-z (PMC8121845; doi:10.1038/s41467-021-22999-z)
Supplement: Supplementary file 1 — Supplementary Information [file 41467_2021_22999_MOESM1_ESM.pdf]

Tuning network topology and vibrational mode localization to achieve ultralow thermal conductivity in amorphous chalcogenides

Kiumars Aryana[1], Derek A. Stewart[2], John T. Gaskins[1], Joyeeta Nag[2], John C. Read[2], David H. Olson[1], Michael K. Grobis[2], and Patrick E. Hopkins*[1,3,4]

[1]Department of Mechanical and Aerospace Engineering, University of Virginia, Charlottesville, Virginia 22904, USA

[2]Western Digital Corporation, San Jose, CA 95119, USA

[3]Department of Materials Science and Engineering, University of Virginia, Charlottesville, Virginia 22904, USA

[4]Department of Physics, University of Virginia, Charlottesville, Virginia 22904, USA

*Corresponding Author: phopkins@virginia.edu

Supplementary Note 1

SiTe atomic structure; amorphous or polycrystalline? The thermal properties of materials are largely dependant upon their atomic structure, specifically, ordered vs.∼ disordered. For instance, it has been shown that the thermal conductivity of crystalline GeTe is almost an order of magnitude higher than the amorphous phase [1, 2]. Given that, tellurium is an element with low glass transition temperature and as a result, compositions with high Te content are prone to crystallization during the deposition process. In current study, the crystallization of the pure Te sample, prompted us to investigate the structure of SiTe samples with high Te content. In order to ensure that the *a*-SiTe and *a*-SeTe films studied here have maintained amorphous structure after the deposition, we perform X-ray diffraction (XRD) and transmission electron microscopy (TEM) on several samples with high Te contents. However, considering the films thickness (<40 nm) and the low glass-transition temperature for these samples (400-500 K), implementing atomic-scale structural characterization without damaging them is exceedingly challenging. Although our XRD measurements for the high Te content samples do not show any substantial peaks that indicate crystallinity, our TEM and EELS micrographs for all SiTe composition except $Si_{11}Te_{89}$ composition, depict Te rich crystallites on the order of 2-5 nm embedded in an amorphous SiO_x matrix. Observations of oxygen signatures and phase segregation in our SiTe films are perplexing. The crystallization could be the result of ion bombardment during the sample preparation process. This is not an unreasonable speculation especially when during TEM imaging/mapping, we observe the electron beam causes further segregation in the region of mapping (see Supplementary Fig. 12), which indicates that the electron beam has sufficient energy to induce phase transformations. Apart from this, we need to mention that although our TEM results indicate an entirely different morphology between $Si_{11}Te_{89}$ (uniform, homogeneous amorphous structure) and $Si_{19}Te_{81}$ (segregated regions with Te rich crystallites in an amorphous SiO_x matrix), their measured thermal conductivity value is identical. This is another indication that the films might have been damaged during the sample prep process. In short, despite the fact that we observe segregation and ordered regions in some of the SiTe samples, we posit that these are artifacts of sample preparation and are not intrinsic to the films.

X-ray diffraction. Table 1 shows the structural details of the films studied in this paper. The compositions and thicknesses are determined by XRF and TEM, respectively. Supplementary Figure 1 (a) shows the result of our XRD measurements for as-deposited pure Te and *a*-$Si_{25}Te_{75}$ with a SiO_2 substrate and 3-5 nm carbon capping layer. We included XRD measurement of *a*-$Si_{17}Te_{83}$ form literature for comparison [3]. For bulk *a*-Si_xTe_{100-x} alloy ($10 \leq x \leq 28$), it was shown that after annealing for 2 hours at 494 K, excess Te starts to crystallize while remaining material stays amorphous. Then, upon annealing at 575 K for another 2 hours, the remainder of amorphous phase crystallize into hexagonal $SiTe_2$ phase [3]. According to this study, the XRD patterns across all compositions for $10 \leq x \leq 28$ are identical. For comparison, we included the result

Supplementary Table 1. The SiTe compositions studied here with the corresponding deposition technique and thicknesses.

Nominal composition	Deposition	Composition Si% XRF (± 3%)	Composition Te% XRF (± 3%)	Thickness TEM (± 1 nm)	Structure TEM
Te	co-sputter	0	100	30	polycrys
$Si_{10}Te_{90}$	co-sputter	11	89	30	amor
$Si_{20}Te_{80}$	co-sputter	19	81	44	amor/polycrys
$Si_{20}Te_{80}$	nano-laminate	38	62	41	amor/polycrys
$Si_{30}Te_{70}$	co-sputter	34	66	37	amor/polycrys
$Si_{40}Te_{60}$	nano-laminate	53	47	41	amor/polycrys
$Si_{50}Te_{50}$	nano-laminate	56	44	47	amor/polycrys
$Si_{70}Te_{47}$	nano-laminate	65	36	45	amor/polycrys
Si	co-sputter	100	0	26	amor

of this study for $Si_{17}Te_{83}$ in Figs. 1(a) and (b). The results in Supplementary Fig. 1(a) is consistent with our expectation. The peaks for pure Te sample agree well with the 494 K annealed sample where only Te has been crystallized. On the other hand, for $Si_{25}Te_{75}$ sample, we do not observe any sharp peak that is indicative of crystalline regions. We repeated this measurement for another set of samples that were prepared for our thermal conductivity measurement with 80 nm of ruthenium coating and the results are presented in Supplementary Fig. 1(b). In these samples, due to the existence of an 80 nm Ru coating and Si substrate, the Te peaks are not completely captured in the XRD data. Here, the only peak that appears in the XRD measurement is at ∼23 degrees. The other peaks in the XRD spectra (labeled accordingly) belongs to Ru transducer and Si substrate [4–6]. Supplementary Figure 2 shows the diffraction pattern for three SiTe compositions with thermal conductivity of 0.1 W m^{-1} K^{-1}. All the samples except for the a-$Si_{30}Te_{70}$ are deposited in a single run. From these results, we conservatively conclude that the as-deposited SiTe films remain amorphous after the deposition. However, to ensure the accuracy of our results we perform transmission electron microscopy.

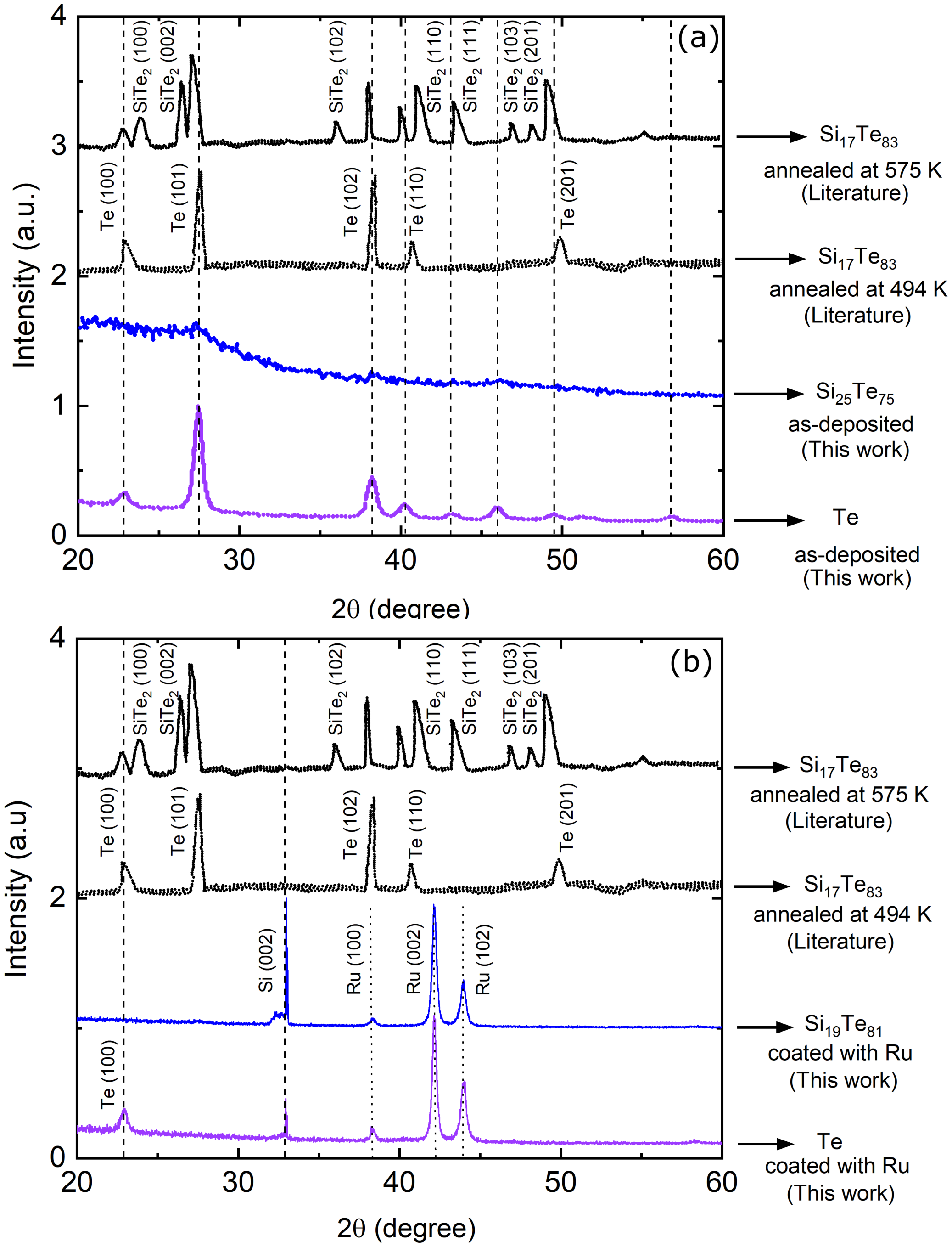

Supplementary Figure 1. Comparison between X-ray diffraction in this study and literature. (a) XRD results are from samples with SiO_2 substrate and 3-5 nm carbon coating, (b) XRD results are from samples with Si substrate and 80 nm specular Ru coating.

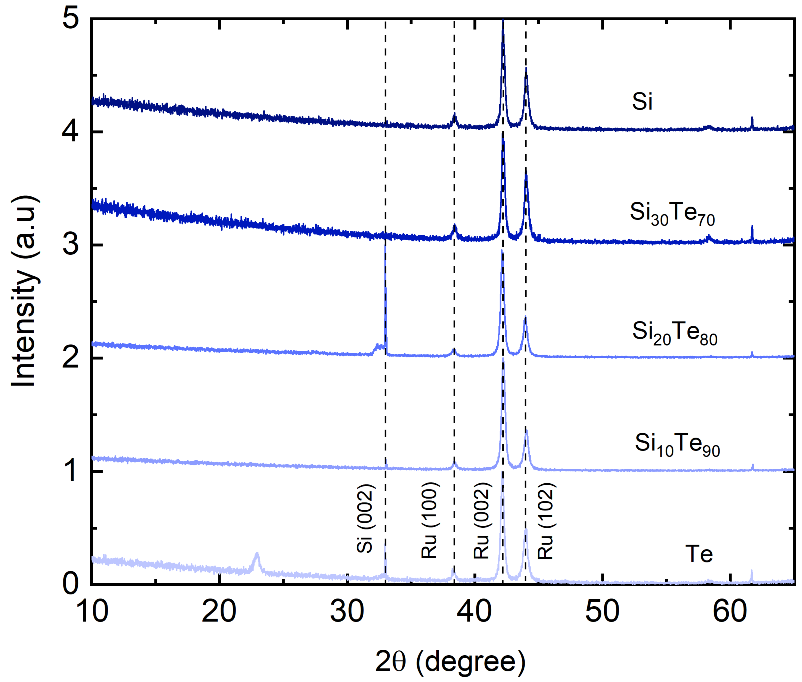

Supplementary Figure 2. Comparison between X-ray diffraction for high Te content samples. The results presented here are for samples with Si substrate and 80 nm specular Ru coating.

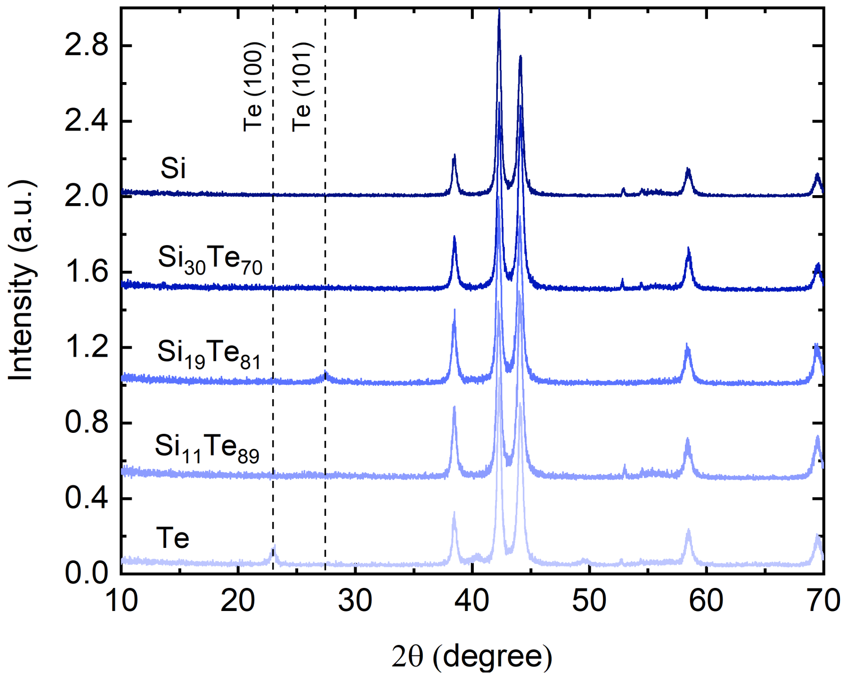

Supplementary Figure 3. Grazing incidence XRD measurements across different compositions of SiTe coated with 80 nm of Ruthenium on a silicon substrate.

Transmission electron microscopy. For this purpose, cross section view Focused Ion Beam (FIB) chips were prepared from the center of each wafer piece. In order to protect the area of interest during sample preparation, the site was coated in the FIB with e-beam SiOx and then tungsten. As part of the FIB sample preparation process, the FIB chip was micro-manipulated onto an Omniprobe stub grid. A JEOL ARM

transmission electron microscope operated at 200 keV was used to collect all images and spectra.

Supplementary Figure 4 shows high resolution TEM results for a-$Si_{11}Te_{89}$ which has the highest concentration of Te amongst our SiTe alloy composition series. For these measurement two different FIB chip were prepared from different parts of the sample and both of the TEM results suggest that the a-$Si_{11}Te_{89}$ film is uniform, homogeneous with amorphous structure. The fact that the silicon substrate maintained its crystal structure is an indicative that the film has not been damaged during sample preparation process.

To further investigate other compositions, we perform STEM EELS maps on samples with nominal compositions of a-$Si_{10}Te_{90}$, a-$Si_{20}Te_{80}$, a-$Si_{30}Te_{70}$, a-$Si_{50}Te_{50}$, and a-$Si_{70}Te_{30}$. As depicted in Figs. 4-9, except a-$Si_{10}Te_{90}$, it appears that all other compositions have segregated into Te rich crystallites surrounded by amorphous SiO_x matrix. This observation is in stark contrast with our expectations due to following reasons. First, the sample deposition occurred in Ar with a few milliTorr pressure where chamber base pressure maintained at mid to low 10^{-9} Torr range, therefore, the existence of oxygen as a result of deposition process is not expected. Second, we have performed depth profiling X-ray photoelectron spectroscopy (XPS) on these samples and no oxygen signature have been found below the Ru layer. Third, as depicted in Supplementary Fig. 4 the samples with the highest concentration of Te, a-$Si_{10}Te_{90}$, maintains its amorphous structure after the deposition. Although, we do not conclusively reject the possibility of phase segregation and emergence of crystallite regions in the SiTe films, considering the XRD measurements, we believe the phase segregation and formation of crystalline regions is an artifact of sample preparation and not intrinsic to the film. In conclusion, although there is a discrepancy between the TEM results where for a-$Si_{10}Te_{90}$ the micrographs show uniform amorphous layer while for a-$Si_{20}Te_{80}$ show a segregated film with crystallite regions, the thermal conductivity for both cases are similar. In addition, the XRD measurements for a-Si_xTe_{1-x} where ($10 \leq x \leq 30$) do not suggest formation of any crystalline regions. All things considered, we believe the films studied in this paper are amorphous or highly disordered polycrystalline. The amorphous-like temperature dependant trend for thermal conductivity of a-$Si_{19}Te_{81}$ further supports our conclusion.

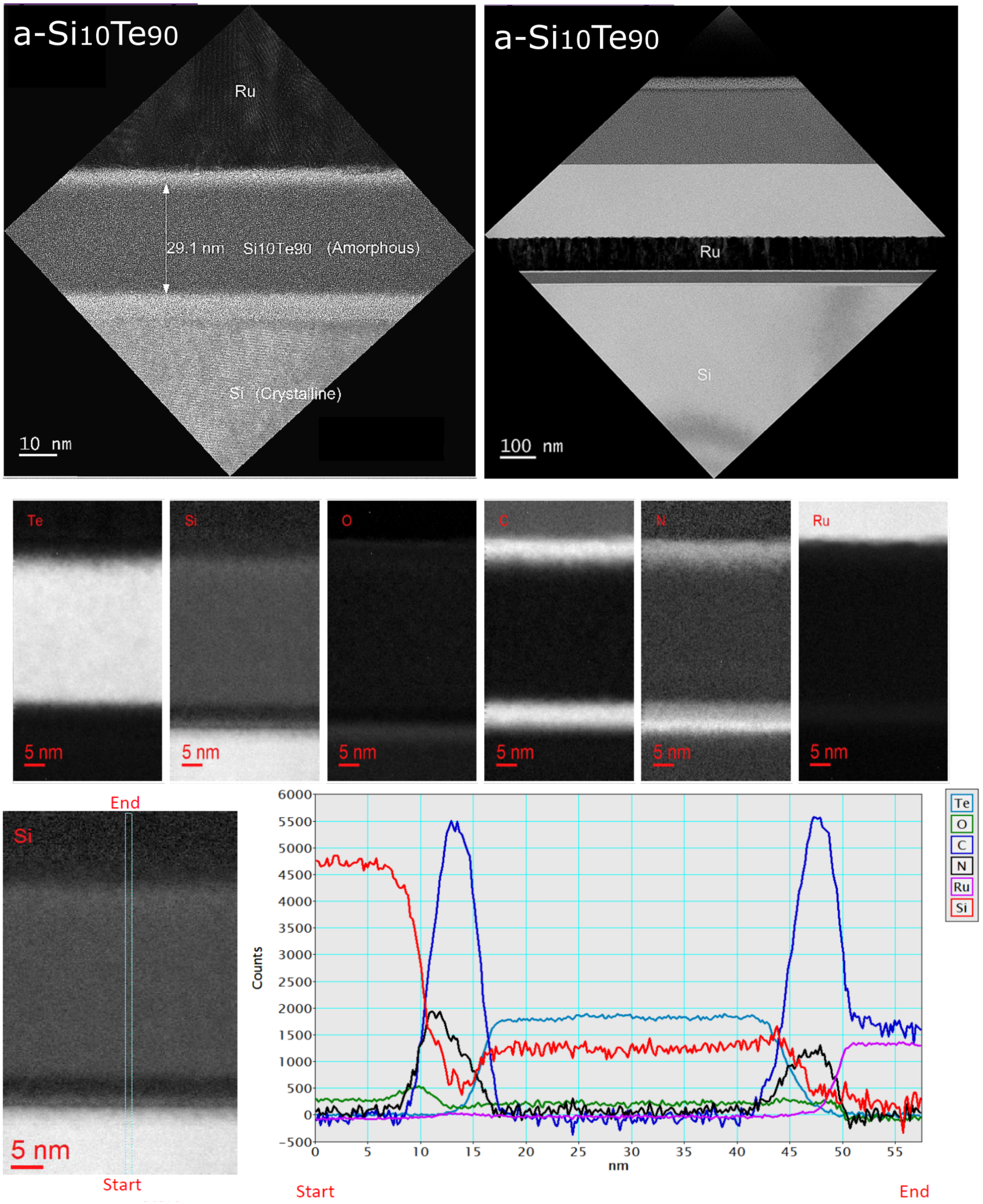

Supplementary Figure 4. TEM micrographs for the sample with nominal composition of *a*-$Si_{10}Te_{90}$. According to these results the film is uniformly amorphous. Little oxygen is in *a*-$Si_{10}Te_{90}$, however, the oxygen level in the *a*-$Si_{10}Te_{90}$ is similar that in Si substrate, so the oxygen could be from air exposure after FIB-cut. Nitrogen in CNx is enriched to the interface of CNx/Si-substrate.

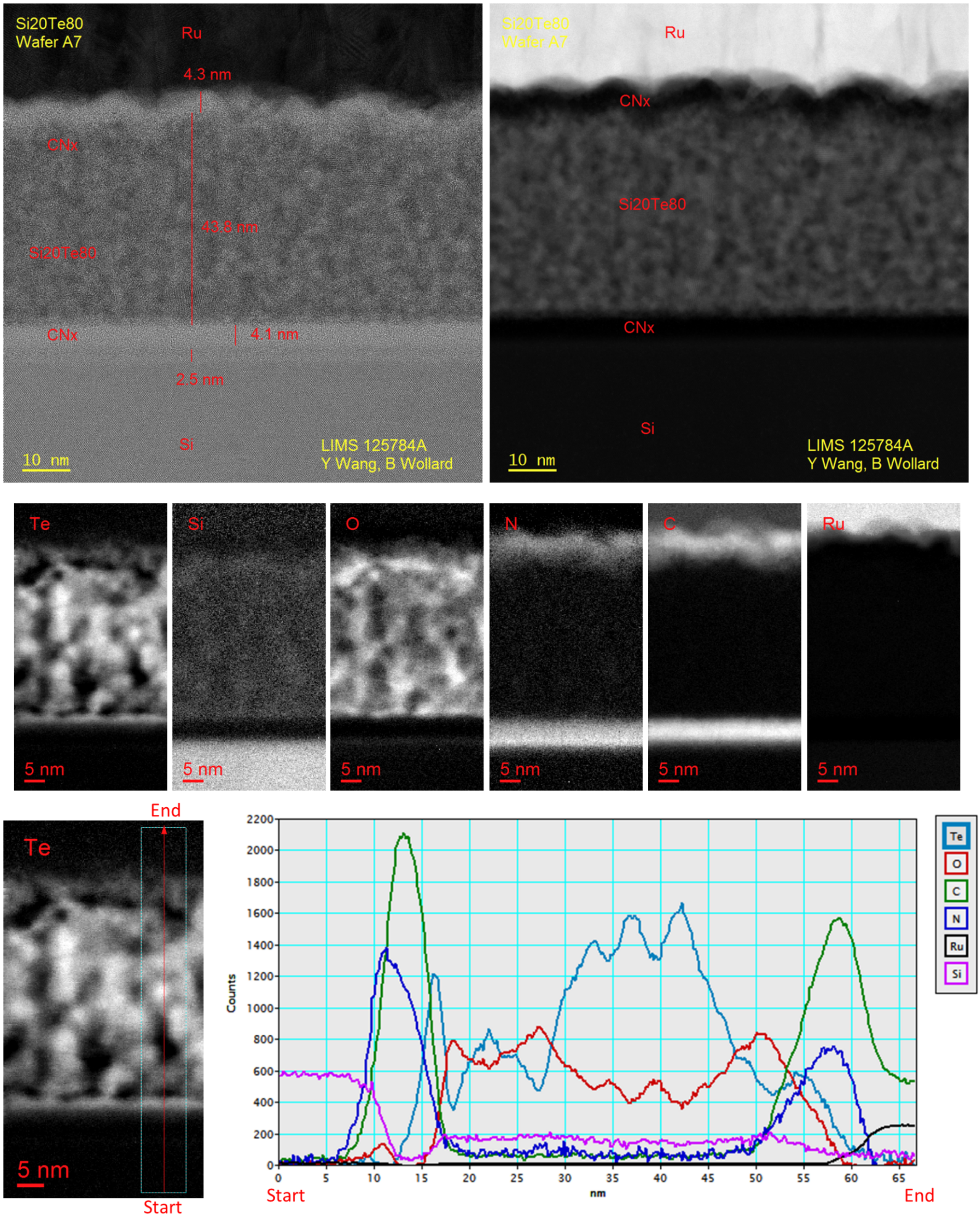

Supplementary Figure 5. TEM micrographs and its corresponding EELS mapping for *a*-$Si_{20}Te_{80}$.

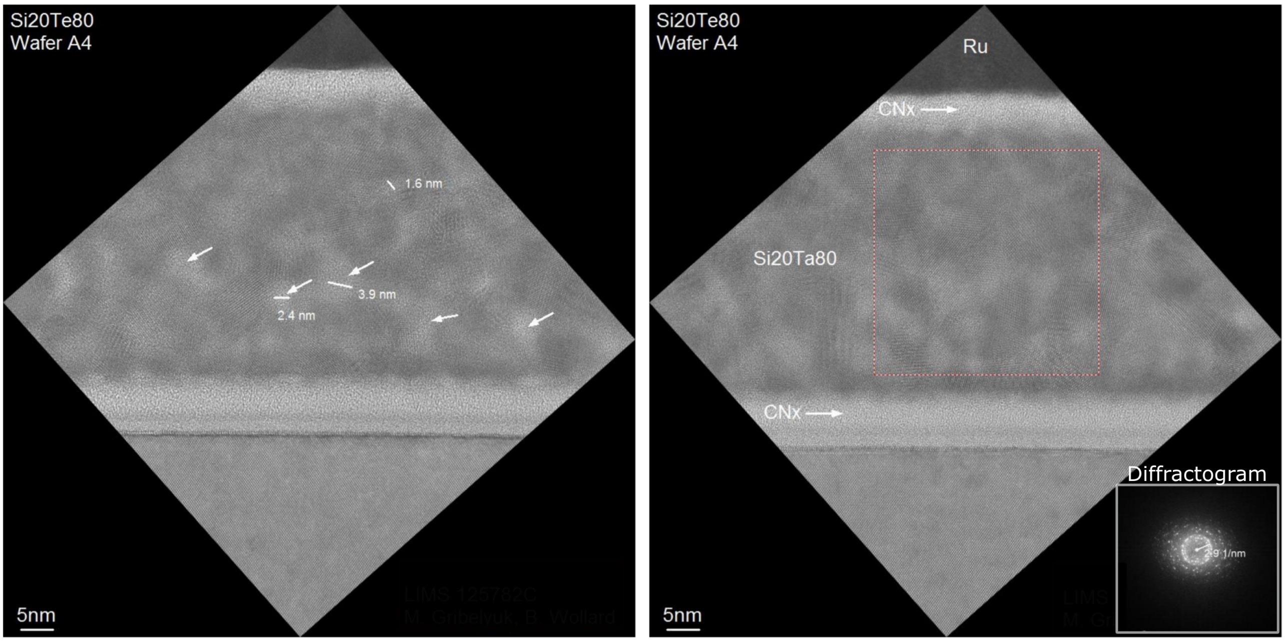

Supplementary Figure 6. TEM micrographs for a-$Si_{20}Te_{80}$, measured for a second time from a different region of the wafer. According to these results the film is compositionally non-uniform. Regions with bright contrast are 1.6nm to 4nm large, appear mostly amorphous. Darker regions are polycrystalline. The prevalent lattice spacing is 3.4A (see diffractogram from selected SiTe region).

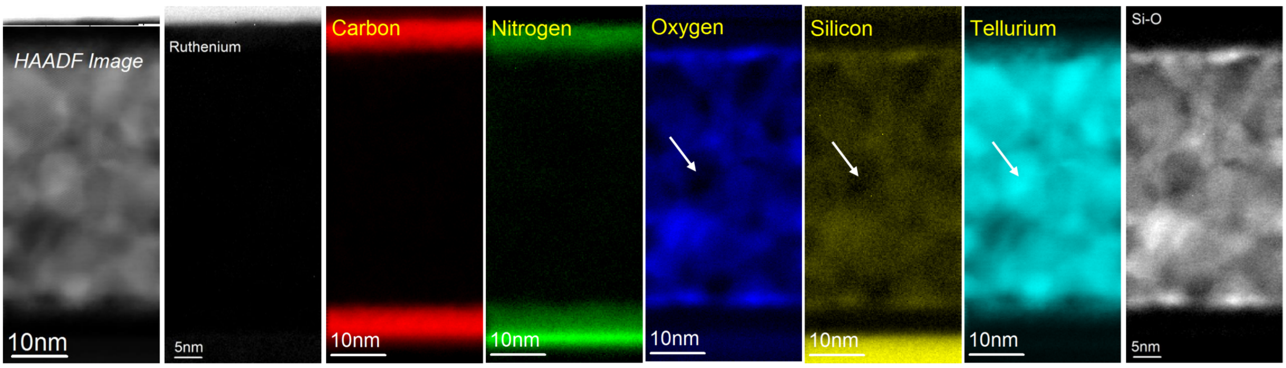

Supplementary Figure 7. TEM micrographs and its corresponding EELS mapping for a-$Si_{20}Te_{80}$.

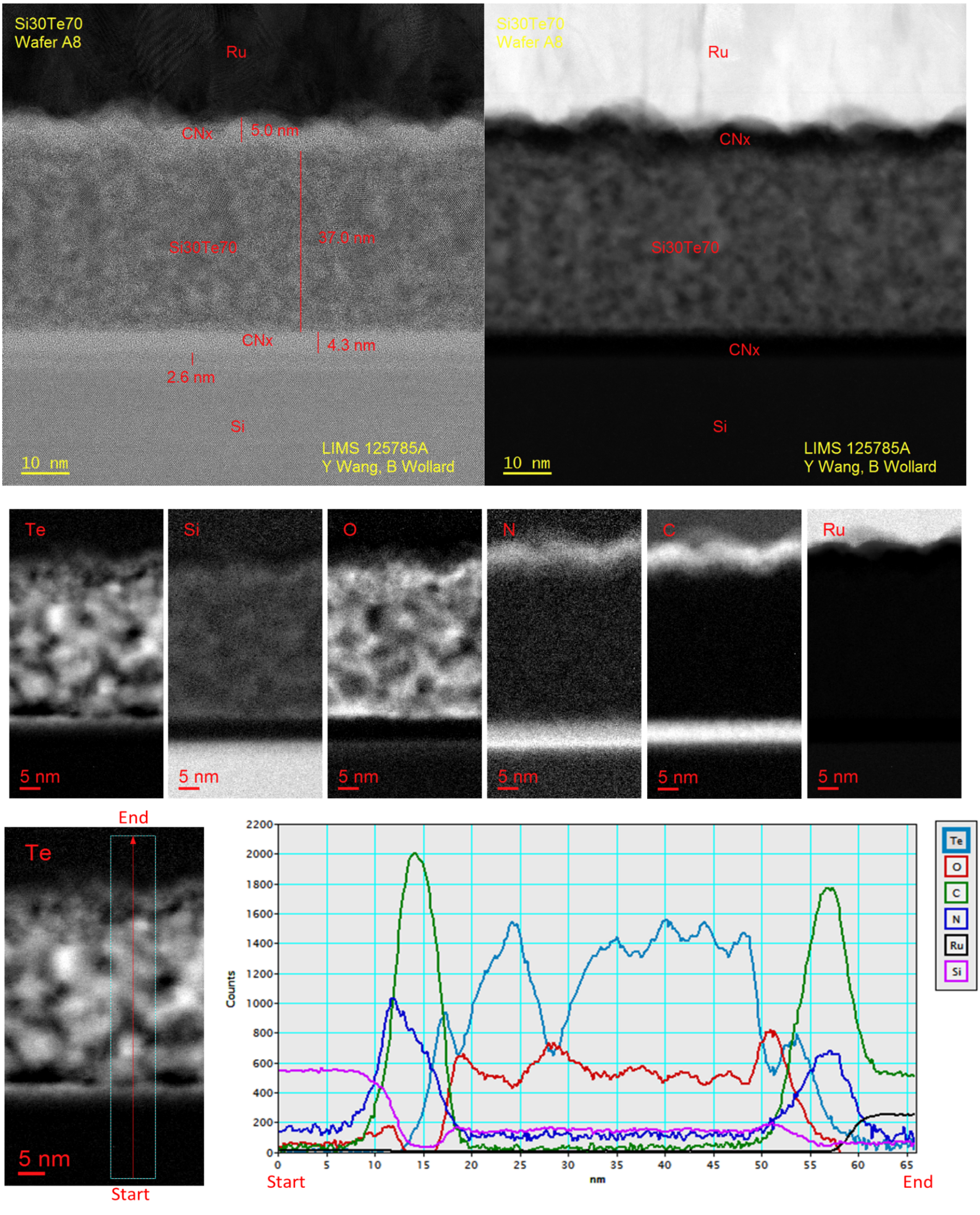

Supplementary Figure 8. TEM micrographs and its corresponding EELS mapping for *a*-$Si_{30}Te_{70}$.

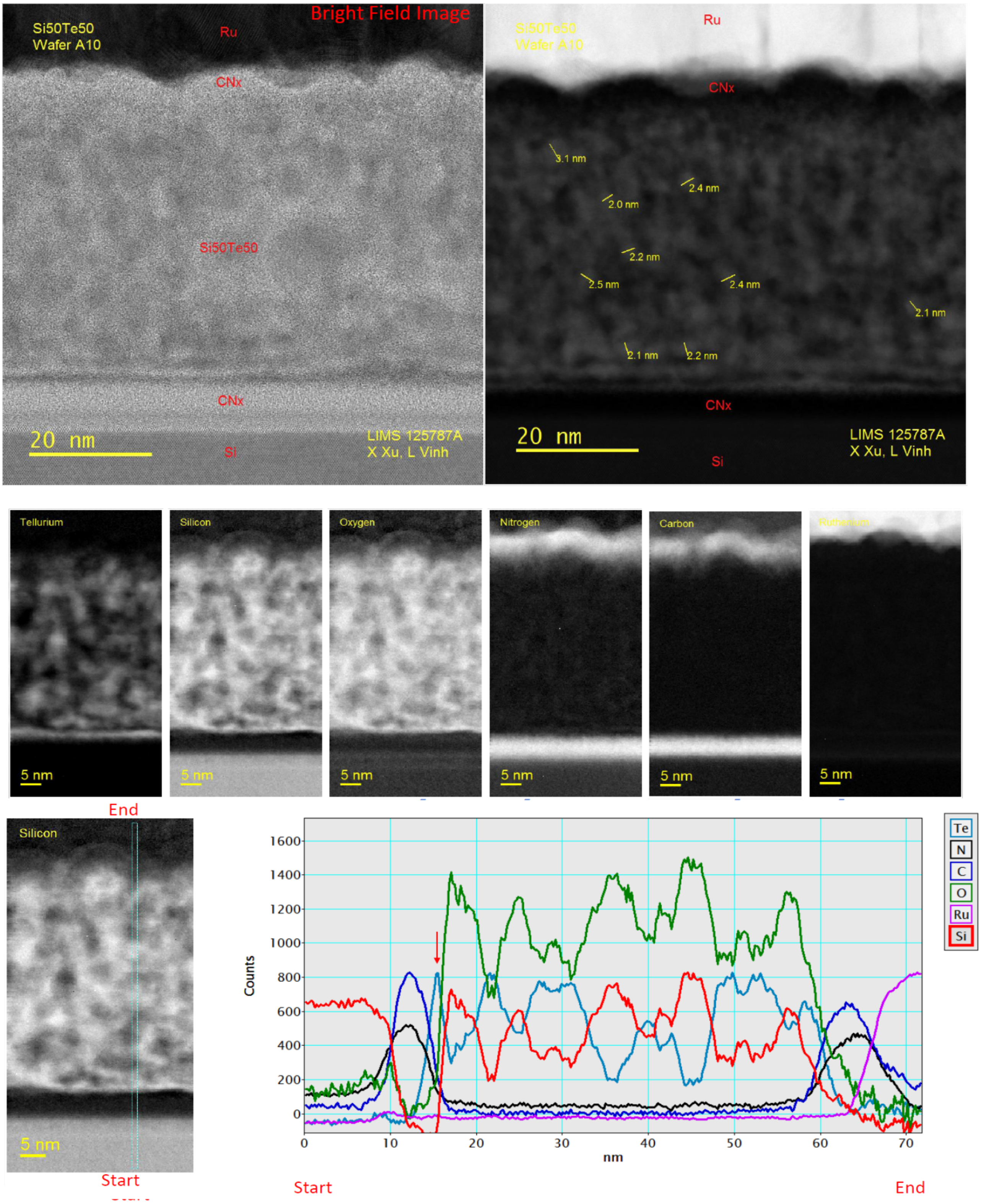

Supplementary Figure 9. TEM micrographs and its corresponding EELS mapping for *a*-$Si_{50}Te_{50}$.

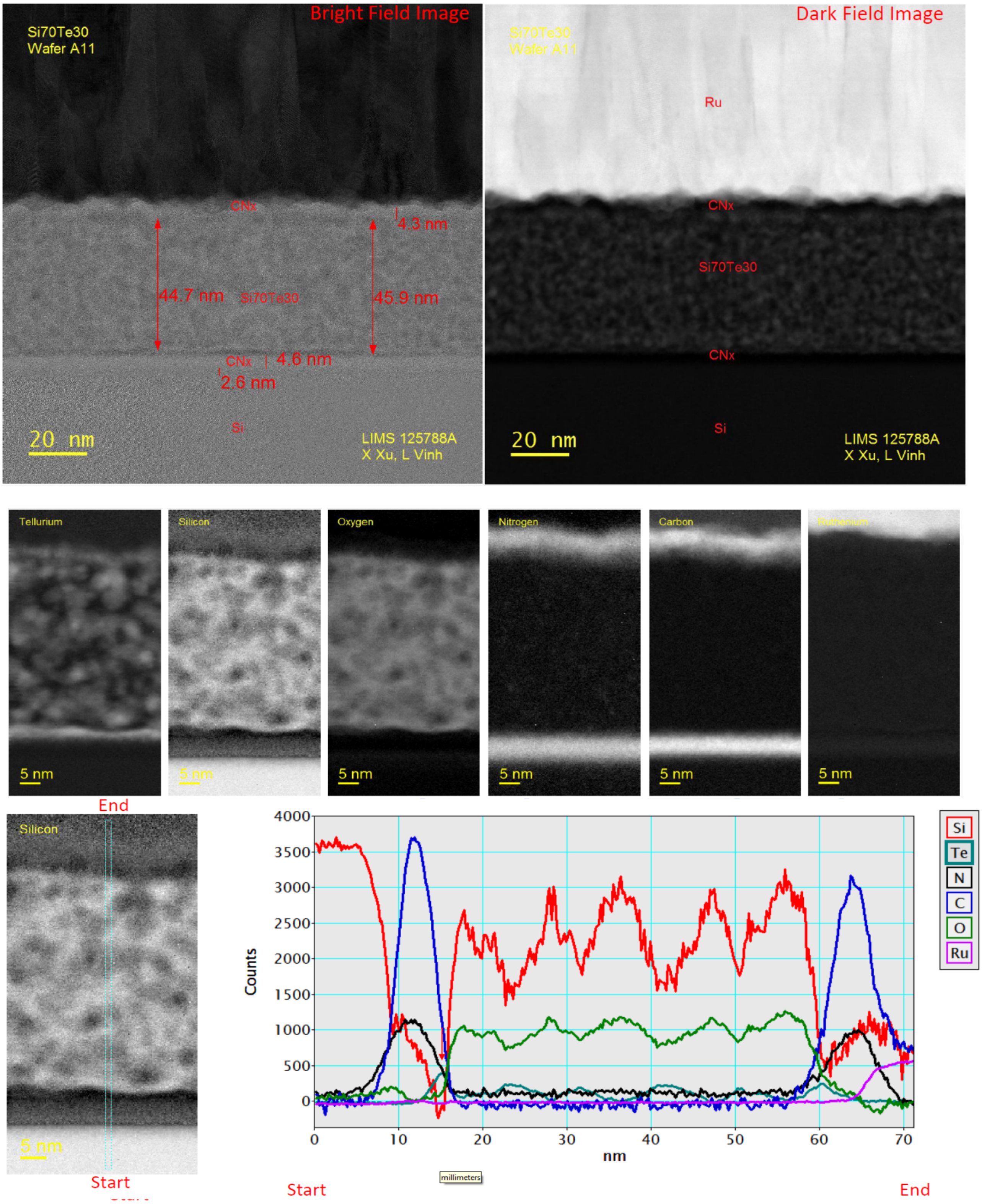

Supplementary Figure 10. TEM micrographs and its corresponding EELS mapping for *a*-$Si_{70}Te_{30}$.

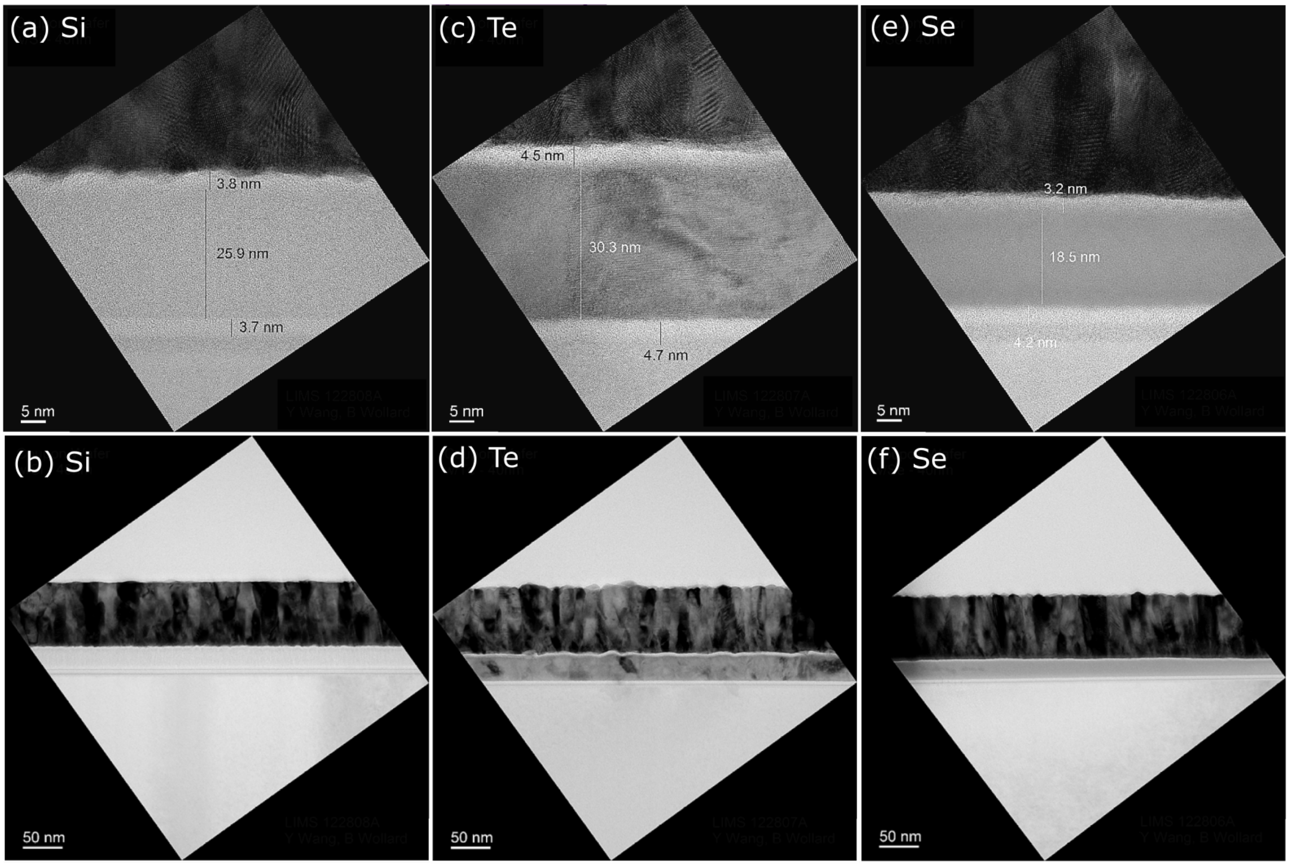

Supplementary Figure 11. TEM micrographs for (a) amorphous Si, (b) polycrystalline Te, (c) amorphous Se.

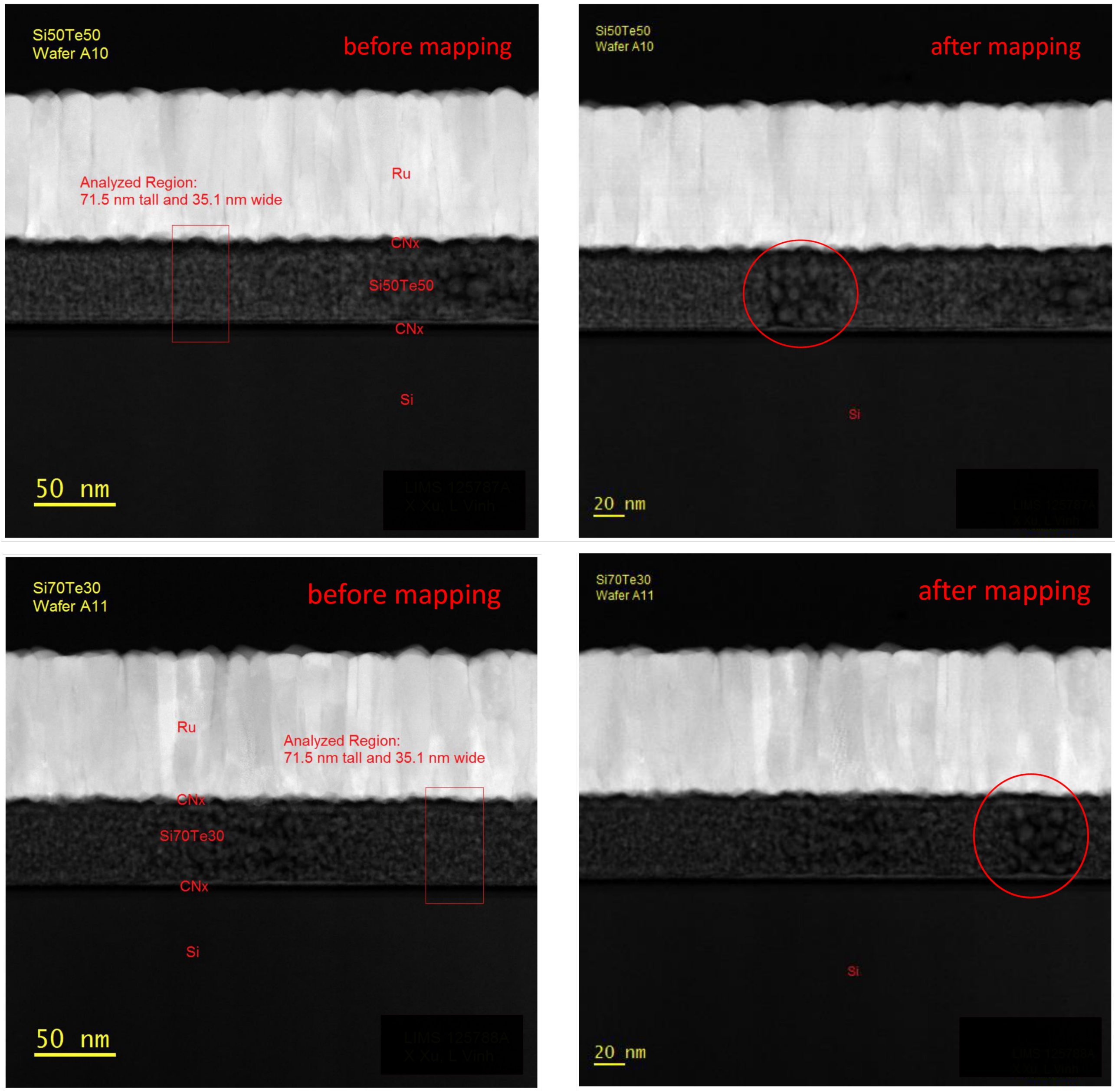

Supplementary Figure 12. TEM micrographs for $Si_{50}Te_{50}$ and $Si_{70}Te_{30}$ before and after mapping. The region specified in red, is where the mapping was performed. As can be seen, SiTe has a high sensitivity to electron beam, and as a result, TEM imaging/mapping causes further segregation in the film. A JEOL ARM transmission electron microscope operated at 200 keV was used to collect all images and spectra.

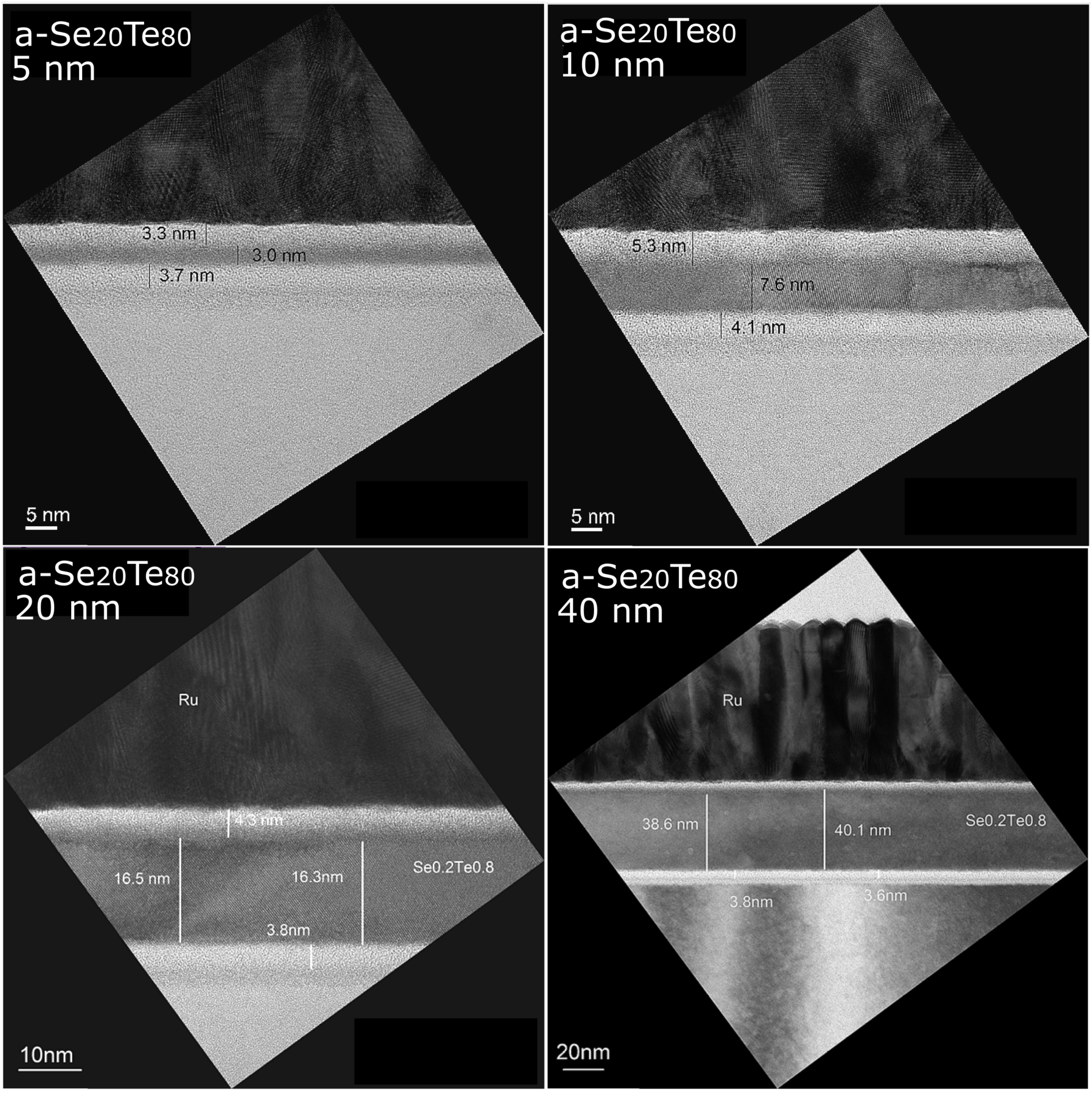

Supplementary Figure 13. TEM micrographs for $Se_{20}Te_{80}$ with nominal thicknesses of 5, 10, 20, and 40 nm. The micrographs for 10 and 20 nm $Se_{20}Te_{80}$ indicate ordered regions in the film. We hypothesise that this crystallinity is not intrinsic to the film and is the result of sample preparation during FIB process.

Raman Spectroscopy. The Raman spectera presented in the main manuscript is taken from the same samples that the thermal conductivity were measured after etching off the 80 nm ruthenium transducer. Due to sensitivity of these films to any external stimuli, the films are prone to damage during the etch off process. Therefore, another batch of samples were prepared without any metal coating for Raman measurement. Figures 14, 15, and 16 show the Raman spectra at different frequency ranges and Te contents for Si_xTe_{1-x} without any metal coating. Although the results for both of these measurements agree well, we believe the results presented here are more representative as there is no metal transducer to interfere with the captured signal and the films are in as-deposited state.

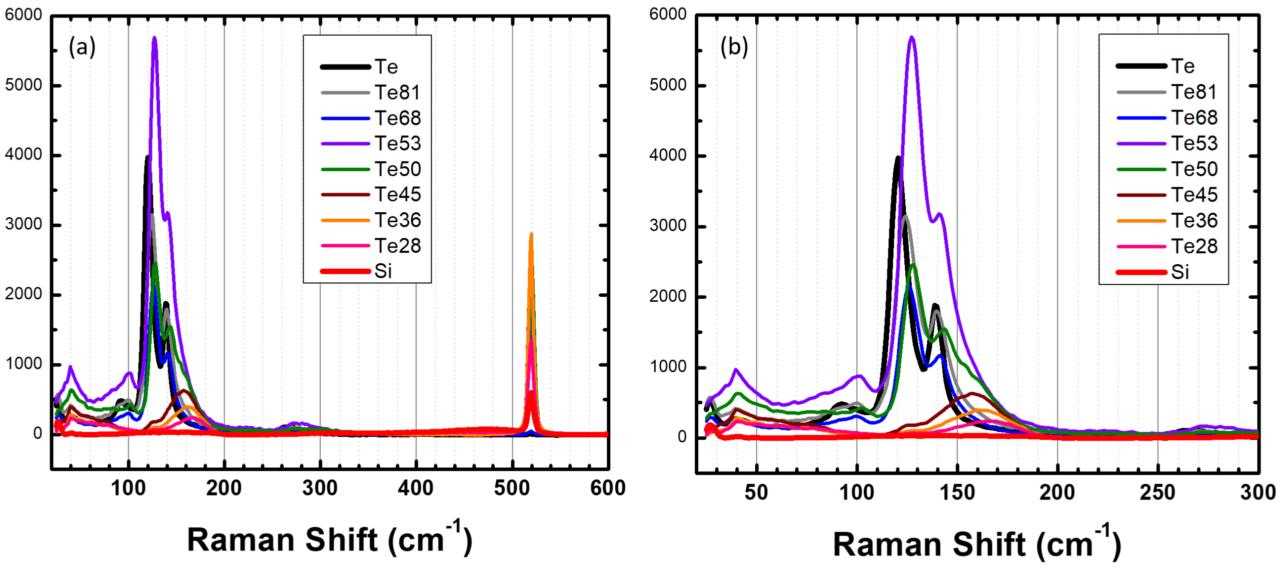

Supplementary Figure 14. The Raman spectra for different compositions of SiTe.

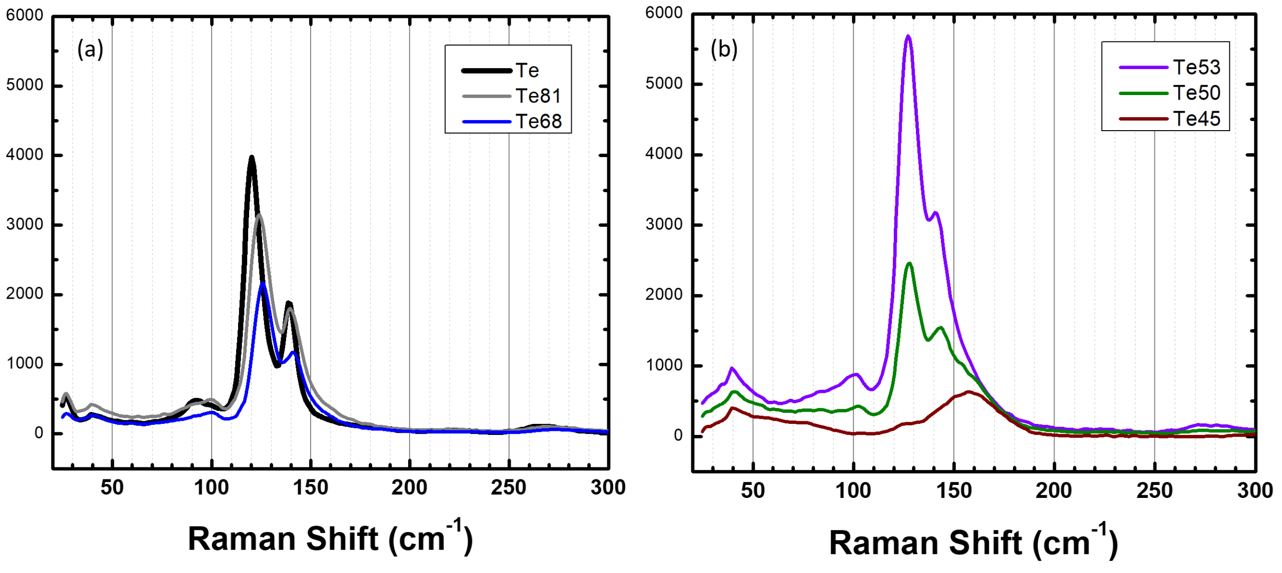

Supplementary Figure 15. The Raman spectra for different compositions of SiTe.

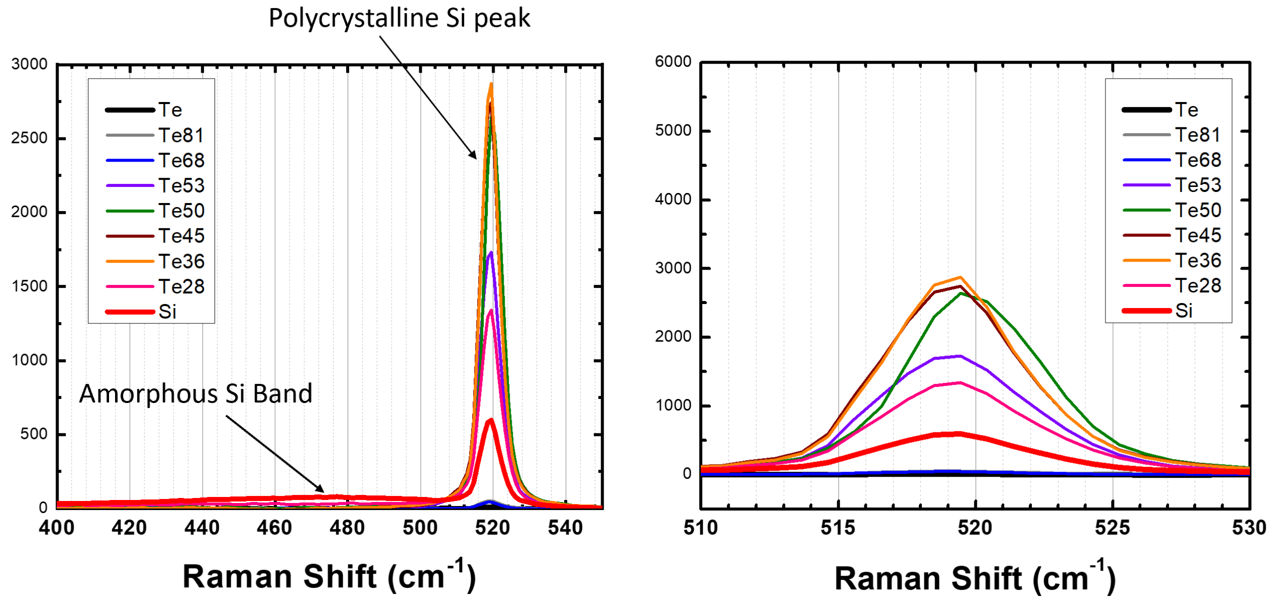

Supplementary Figure 16. The Raman spectra for different compositions of SiTe.

Supplementary Note 2

Thermal conductivity measurements. The thermal conductivities reported in this study are measured using time-domain thermoreflectance (TDTR) in a two-tint setup where the output of an 80 MHz Tsunami Ti:Sapphire oscillator with center wavelength of 808 nm is spectrally separated into a pump and a probe path. The spot sizes for pump and probe beams are ∼20µm and ∼10µm, respectively. The pump path is fixed at modulation frequency of 8.4 MHz via an electro-optic modulator (EOM). The thermal penetration depth ($d_p = \sqrt{k/\pi f_{mod} C}$) at this frequency for the composition with the lowest thermal conductivity ($Si_{20}Te_{80}$) is 54 nm which is well above our thickest SiTe film (40 nm). The thermal conductivity of the Ru transducer (∼54 W m^{-1} K^{-1}) is determined via Wiedemann–Franz law where the electrical resistivity obtained via four-point probe measurements. The temperature-dependant specific heat for Ru transducer is obtained from the literature [7]. The thicknesses for each layer are obtained from transmission electron microscopy (TEM) micrographs.

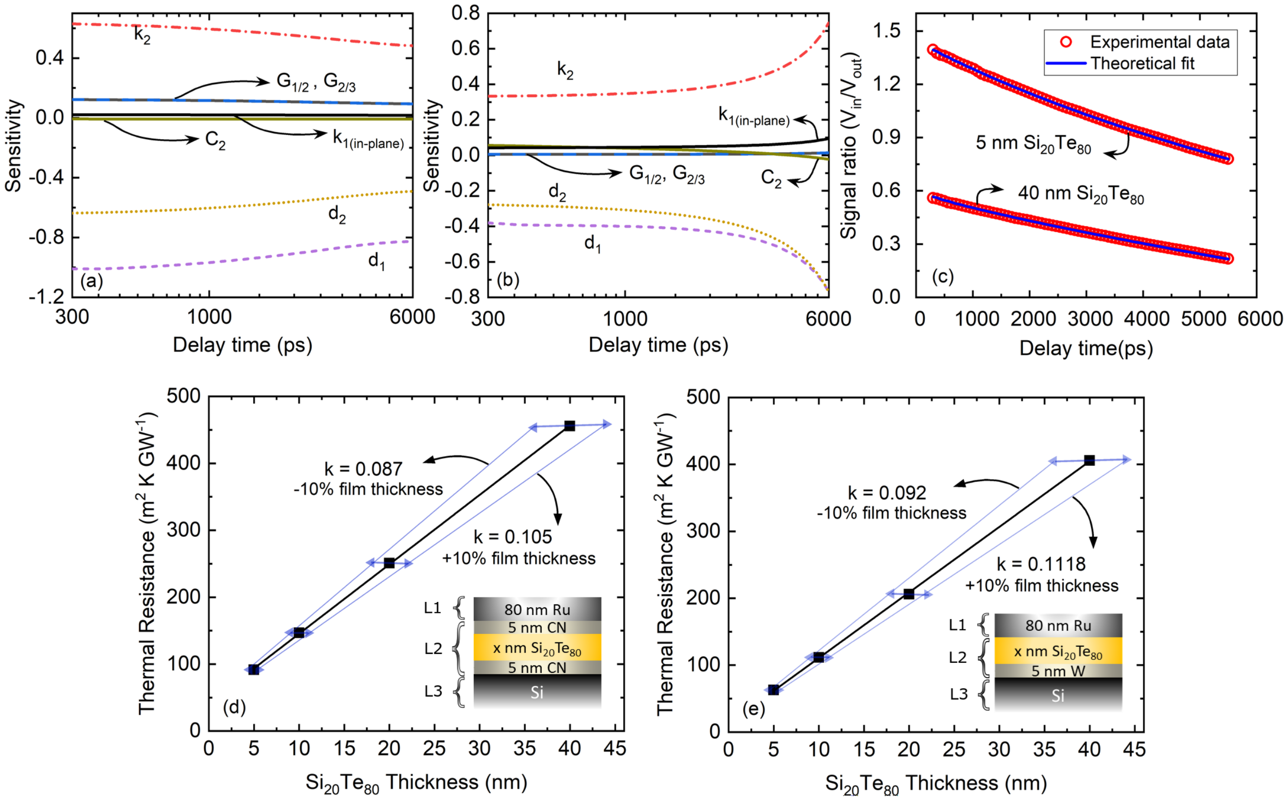

Supplementary Figure 17. Sensitivity of our measurement to thermal conductivity, k, thermal boundary conductance, G, specific heat, H, and layers thickness, d, for (a) 5 and (b) 40 nm thick $Si_{20}Te_{80}$, (c) a representative fit to the experimental data for a 5 and 40 nm thick $Si_{20}Te_{80}$. (d,e) thermal conductivity of $Si_{20}Te_{80}$ for different samples configurations, obtained by applying a linear fit to the thermal resistance as a function of thickness. The solid triangles correspond to uncertainty and is calculated by assuming 10% variations in the $Si_{20}Te_{80}$ film thickness.

For fitting thermal model to the experimental data, we use the ratio of in-phase versus out-of-phase signals ($-V_{in}/V_{out}$). Our thermal model calculations assume that there are 3 effective layers in the direction of heat flow i.e. Ru transducer, CN/SiTe/CN stack, and the substrate (see schematic in Supplementary Figs. 17(d,e)).

Figures 17(a,b) demonstrate the sensitivity of our analysis to parameters such as thickness (d), specific heat (C), thermal conductivity (k), and the thermal boundary conductance (G) for a 5 and 40 nm thick $Si_{20}Te_{80}$ film, respectively. The subscripts indicate the layer number which are labeled as L1, L2, and L3 in the schematics in Supplementary Figs. 17(d,e). These calculations suggest that we are mostly sensitive to thickness of the first and second layers. The uncertainty is estimated by varying the thickness of the SiTe film (d_2) by 10%. Another important parameter that needs to be taken into account for estimating the thermal conductivity of the second layer, is thermal boundary conductance (TBC) at the interfaces. As can be seen in Supplementary Fig. 17(a), the sensitivity of our analysis to the thermal conductivity of $Si_{20}Te_{80}$, even for a 5 nm thick film is much higher than the thermal boundary conductance. Therefore, in our analysis, we assume infinite TBC for the $G_{1/2}$ and $G_{2/3}$ interfaces and only fit for the thermal conductivity of the $Si_{20}Te_{80}$ layer which is the main source of thermal resistance in the stack. Using a separate set of samples, we measure the thermal conductance across Ru/ 10 nm CN/Si and subtract the resistance from the layer thermal conductivity to account for the boundary resistances. However, since the resistance of the $Si_{20}Te_{80}$ is large, subtracting the boundary conductance from the layer has negligible effect on the measured value. A representative experimental data and its corresponding fit is demonstrated in Supplementary Fig. 17(c) for a 5 and 40 nm thick $Si_{20}Te_{80}$ film. As can be seen, although we assume unrealistic values for the TBC, due to negligible sensitivity, the model perfectly fits to the experimental data. To ensure the accuracy of our measurements for thermal conductivity, we calculate the total thermal resistance between Ru and Si layer for different film thicknesses. Then, by applying a linear fit to the thermal resistance data, we obtain the thermal conductivity that is independent of the TBCs. We apply this to 2 different sample configurations with different interlayers to ensure the accuracy of our measurements and negligible impact of TBC on our thermal conductivity measurements. Based on these two sample configurations, we measure the thermal conductivity of the $Si_{20}Te_{80}$ film to be 0.1 ± 0.01 W m^{-1} K^{-1}, in perfect agreement with our 3 layer thermal model fit for the 40 nm thick film. The uncertainty is calculated by varying the thickness of the film by $\pm 10\%$. In Supplementary Figs. 18 and 19, we demonstrate our thermal conductivity measurements for SeTe at two different composition $Se_{50}Te_{50}$ and $Se_{20}Te_{80}$ and their corresponding TEMs.

Supplementary Figure 20 shows the thermal conductivity of $Si_{20}Te_{80}$ at elevated temperature before the sample starts to delaminate. For these measurements, we use a resistive heating stage with nitrogen gas flow and increment the temperature of the sample every 10 minutes by 20°. We do not observe any changes in the thermal conductivity of $Si_{20}Te_{80}$ at elevated temperature.

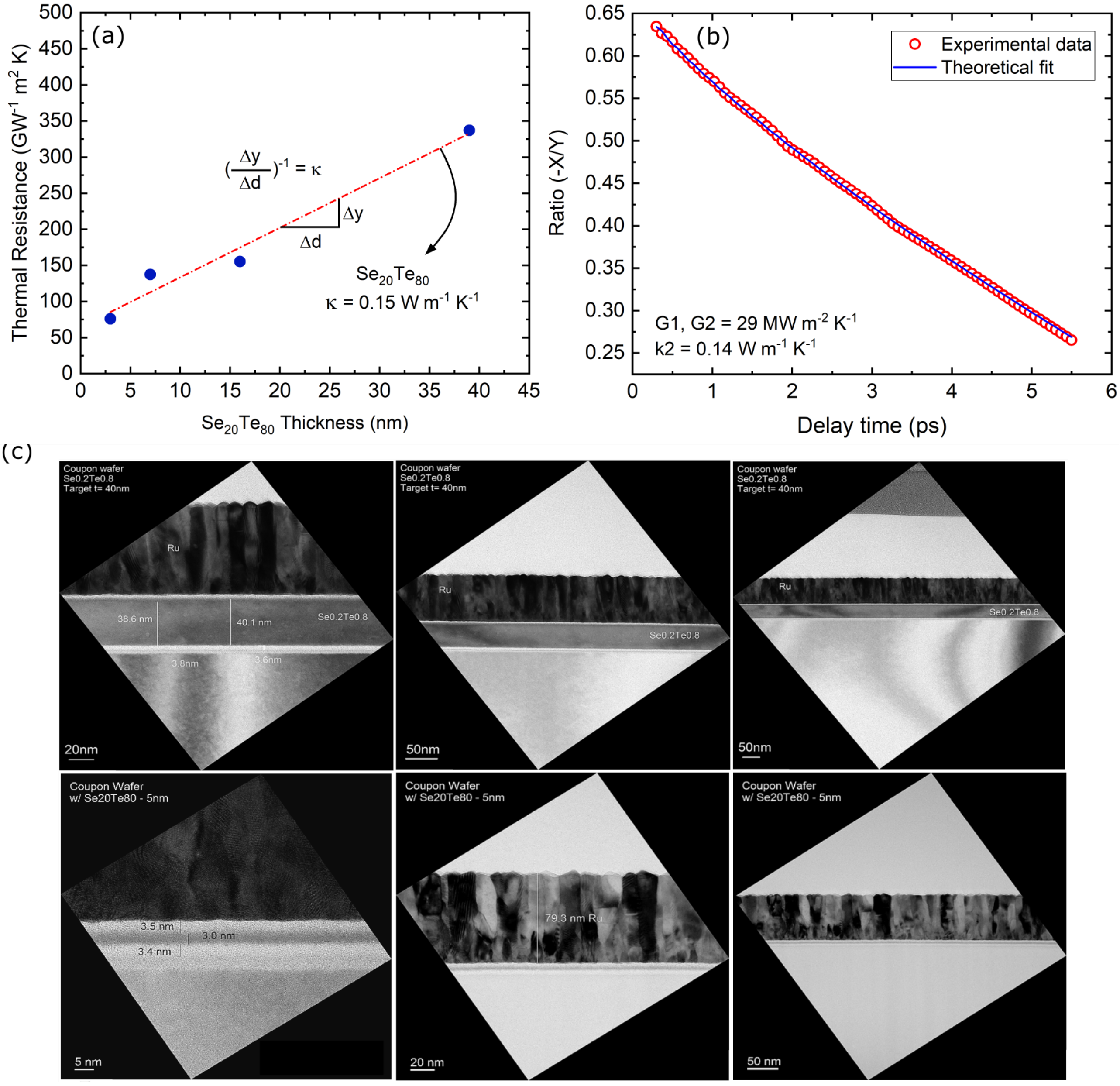

Supplementary Figure 18. (a) Thermal resistance across Ru/CN$_x$/Se$_{20}$Te$_{80}$/CN$_x$/Si layers as a function of SeTe film thickness. The inverse of slope for the linear fit corresponds to the intrinsic thermal conductivity of the Se$_{20}$Te$_{80}$. (b) A representative theoretical fit to the experimental data for ∼40 nm of Se$_{20}$Te$_{80}$, (the only fitting parameter is thermal conductivity of Se$_{20}$Te$_{80}$ film, k2) and (c) the corresponding TEMs at different magnifications.

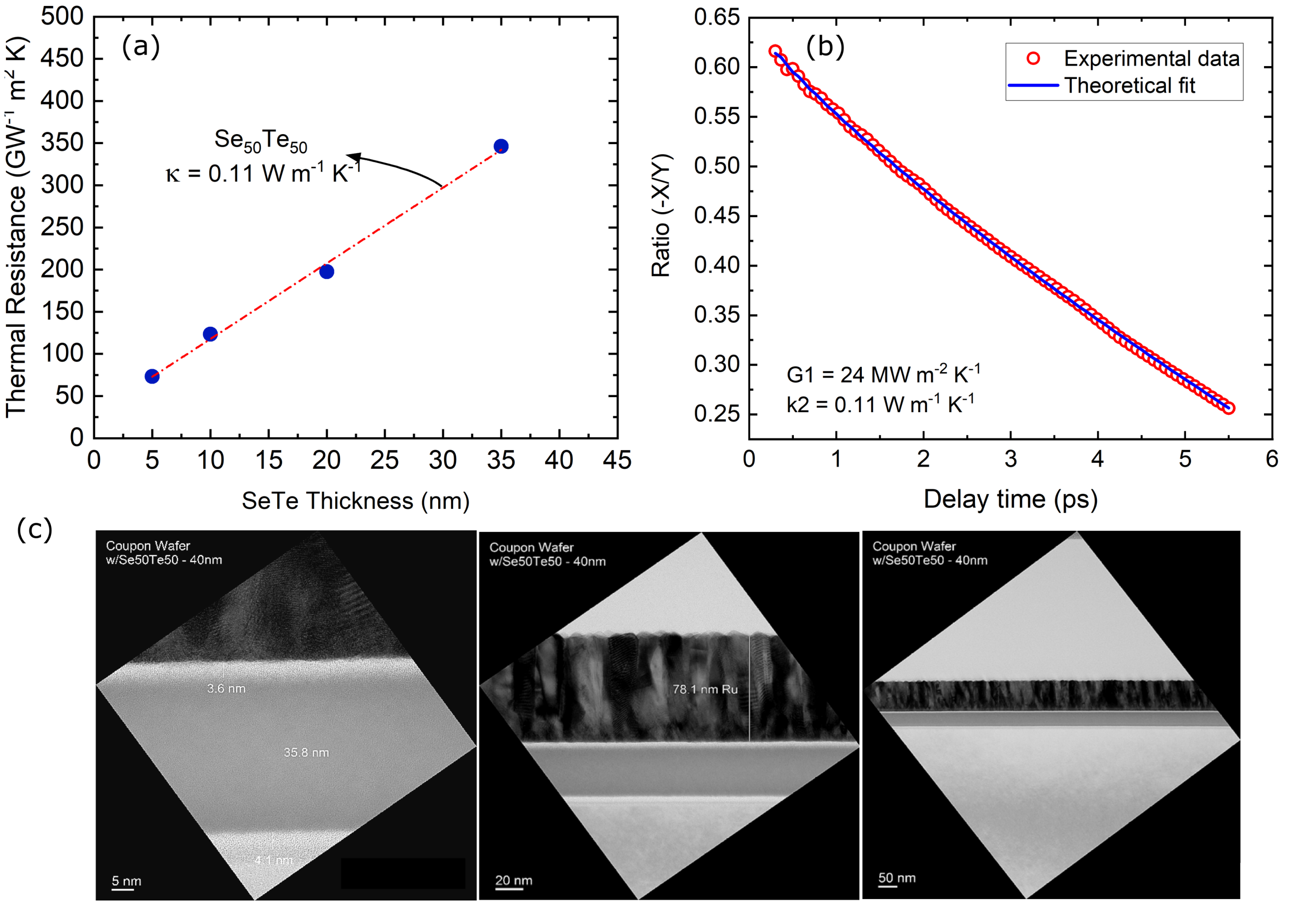

Supplementary Figure 19. (a) Thermal resistance across Ru/CN$_x$/Se$_{50}$Te$_{50}$/CN$_x$/Si layers as a function of SeTe film thickness. The inverse of slope for the linear fit corresponds to the intrinsic thermal conductivity of the Se$_{50}$Te$_{50}$. (b) A representative theoretical fit to the experimental data for 35.8 nm of Se$_{50}$Te$_{50}$, and (c) the corresponding TEMs at different magnifications.

Supplementary Table 2. The lowest thermal conductivity materials and their corresponding crystal structure.

	Composition	Structure	Thermal Conductivity ($W m^{-1} K^{-1}$)
Chiritescu et al. [8]	WSe_2	layered crystalline	0.05
Duda et al. [9]	PCBM	disordered crystalline	0.055 ± 0.015
Zhang et al. [10]	$Ge_{20}Te_{72}Se_8$	amorphous	0.095
This work	$Si_{20}Te_{80}$	amorphous	0.10 ± 0.01
Rasel et al. [11]	$[(C_n H_{2n-1} NH_3)_2PbI_4$	single-crystalline	0.099-1.25
Giri et al. [12]	$isoBA_2PbI_4$	layered crystalline	0.10 ± 0.02
Zhao et al. [13]	SnSe	crystalline	0.23 ± 0.03
Lee et al. [14]	$CsSnI_3$	single-crystalline	0.38 ± 0.04

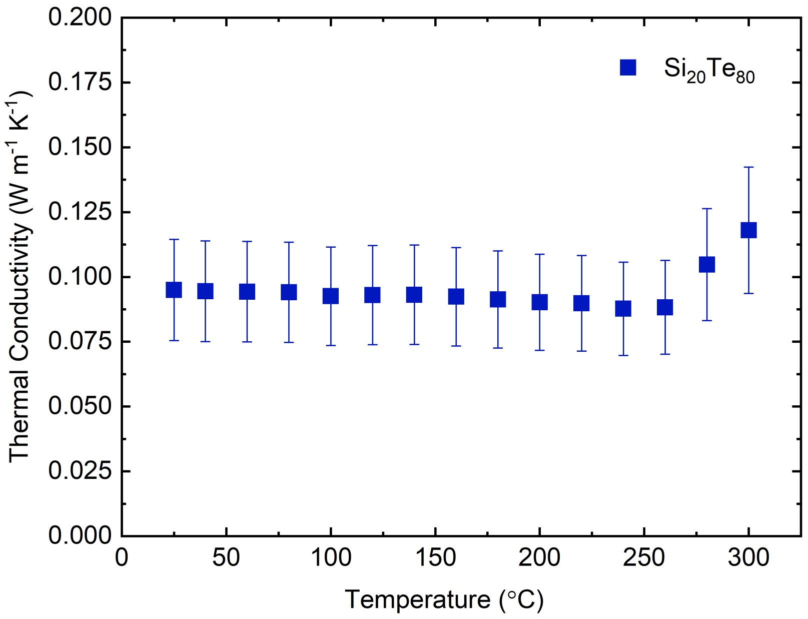

Supplementary Figure 20. Thermal conductivity of 20 nm thick $Si_{20}Te_{80}$ from room temperature up to 300 °C. The film started to delaminate for temperatures above 300 °C. The uncertainty is calculated by assuming 10% variations in the $Si_{20}Te_{80}$ film thickness.

Sound speed measurements. In order to measure the speed of sound in the SiTe films across different compositions, we use picosecond acoustics. In this technique, the absorption of laser pulse on the Ru surface, launches mechanical strain waves from the surface to the underlying layers. The strain waves travel at the speed of sound in the corresponding layer. At the interface between the layers, however, depending on the acoustic impedance defined as ($Z = \rho \times E$) where ρ is density and E is Young's modulus, the waves are partially reflected and the remainder is transmitted. The reflected waves from each interface travels all the way back to the surface and influence the thermoreflectivity of the transducer. For the case where acoustic impedance at the interface is significant and a large portion of the strain waves are reflected, using a picosecond time resolution, the echoes can be detected with the probe beam in the TDTR signal as troughs and peaks. These echoes are only detectable when there are a few number of layers and the acoustic impedance between the layers of interest is large. For instance, for a-Si and Te sample studied here, due to lack of sufficient acoustic impedance at the interfaces, we have not been able to detect any echoes. Therefore, we report the sound speed for these two samples from the literature [15]. On the other hand, as can be seen from the solid line in Supplementary Fig. 21, the acoustic impedance at the Ru/Si interface is sufficiently large to influence our TDTR signal. In this case, the troughs corresponds to the reflected waves from the Ru/Si interface. Addition of $CN_x/Si_{20}Te_{80}/CN_x$ layers between Ru and Si, depicted as solid circles, as well as changing the decay rate in the TDTR signal, add additional peaks. These peaks correspond to the interface between CN_x/Si. By measuring the time between the first trough corresponding to Ru/CN_x interface and the first peak corresponding to the CN_x/Si, we estimate the time it takes for the strain waves to travel across the $CN_x/Si_{20}Te_{80}/CN_x$ stack. In this case, since the sound speed in CN_x is unknown, it is difficult to deconvolve the sound speed of SiTe from from that of the CN_x.

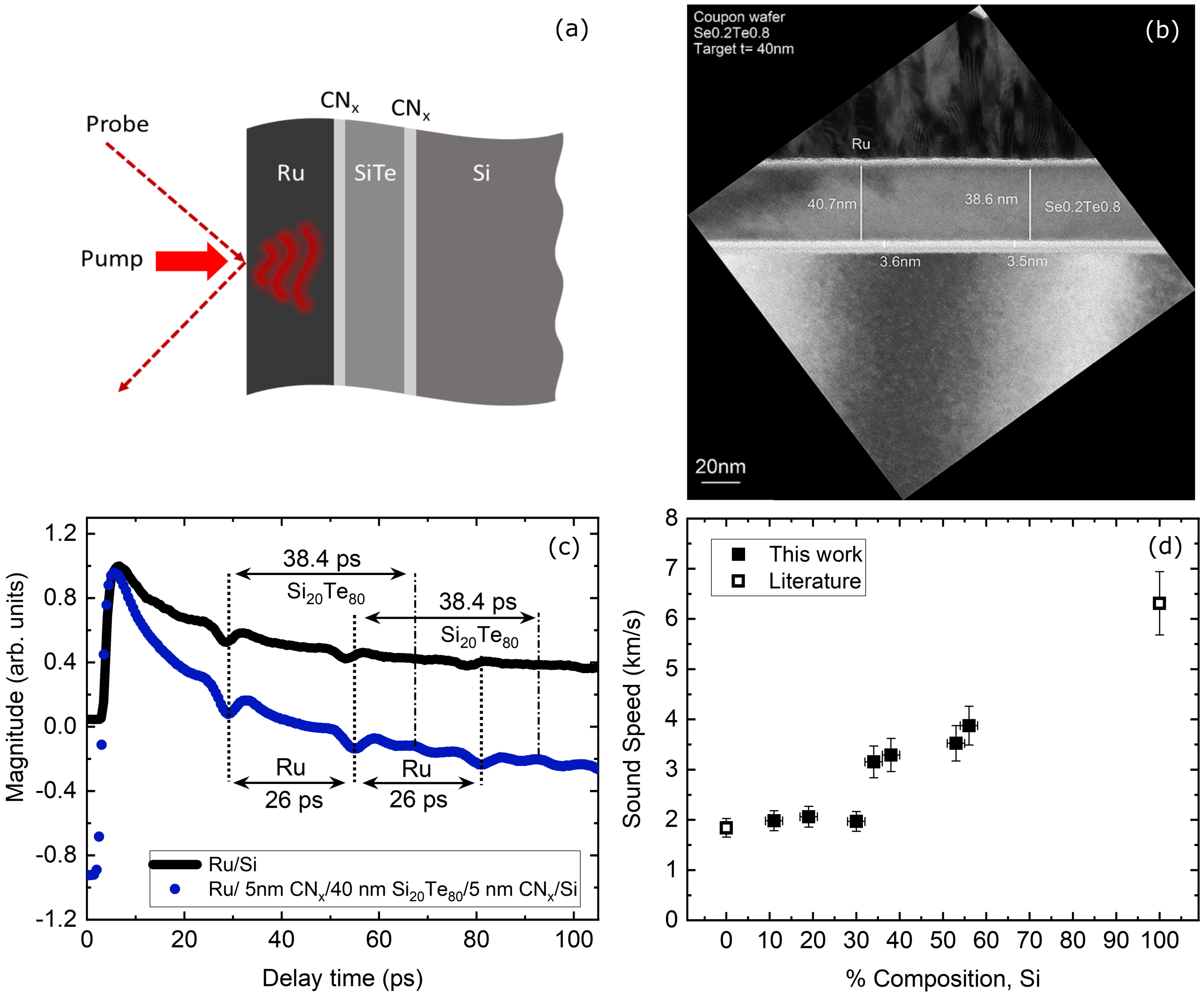

Supplementary Figure 21. (a) Schematic for the layers configuration studied here and (b) the corresponding TEM for 40 nm *a*-$Si_{20}Te_{80}$. (c) TDTR thermal decay signal in picosecond resolution showing the echoes from different interfaces for with and without $CN_x/Si_{20}Te_{80}/CN_x$ stack. (d) The measured sound speed using picosecond acoustics across different compositions. The uncertainty is calculated based on 10% variations in the film thickness.

In order to accurately pinpoint the sound speed in $Si_{20}Te_{80}$ layer, we deposited another batch of samples without the CN_x interlayer: 80 nm Ru/5-40 nm $Si_{20}Te_{80}$/5 nm W/Si (see Supplementary Fig. 22). The W layer between the film and the Si substrate is to ensure sufficient reflection from the backside interface. The measured sound speed from 40 nm thick $Si_{20}Te_{80}$ is 2150 ± 100 m s^{-1}. Using the obtained sounds speed for $Si_{20}Te_{80}$ we estimate the sound speed in CN_x to be 7500 ± 900 m s^{-1} which is well within the range for amorphous diamond like carbon. The nitrogen content in CN_x is nearly 20%.

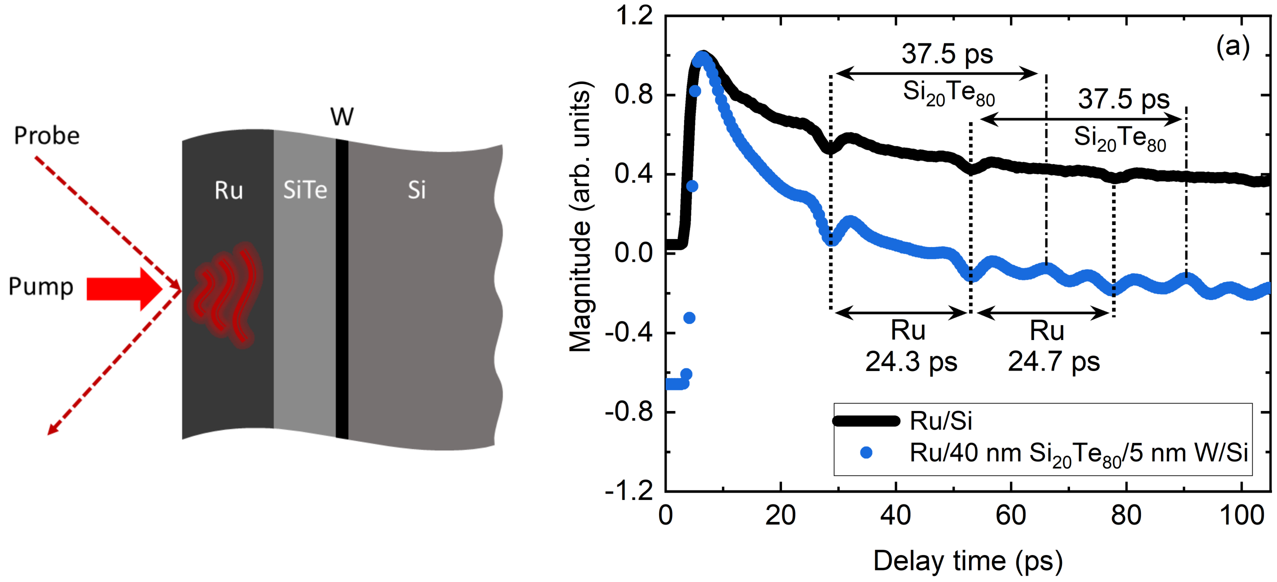

Supplementary Figure 22. (a) Schematic for the layers configuration studied and (b) the TDTR thermal decay signal showing echoes from different interfaces for with and without $Si_{20}Te_{80}$/W stack.

Supplementary Note 3

Molecular dynamics simulations. In order to exclusively investigate the effect of mass scattering on a-SiTe alloy, molecular dynamics (MD) simulations were performed by randomly substituting the mass of Si with that of Te (127 u). We use non-equilibrium molecular dynamics (NEMD) and equilibrium molecular dynamics (NMD) methods to calculate the thermal conductivity of a-Si_{20}-heavySi_{80}. For both techniques, we use Stillinger-Weber interatomic potential that has been widely used to characterize the thermal properties of Si. The simulation procedure and details of these techniques are given elsewhere [16–18]. Supplementary Fig. 23 (a) demonstrates the temperature profile across the simulation box. For these simulations two heat bath are assigned at the beginning and in the middle of the simulation box. The temperature of the hot and cold regions are set to 550 and 450 K, respectively. By measuring the heat flux transferred between these two heat baths, the thermal conductivity of the a-Si_{20}-heavySi_{80} is determined to be 0.50 W m^{-1} K^{-1}. This calculation is in good agreement with our EMD green-kubo calculations as shown in Supplementary Fig. 23 (b). This thermal conductivity is a factor 5 higher than the measured thermal conductivity for a-$Si_{20}Te_{80}$. Therefore, we conclude that although there is a large atomic mass mismatch between Si and Te, the mass scattering alone in a-Si_{20}-heavySi_{80} cannot explain the ultralow thermal conductivity of this material.

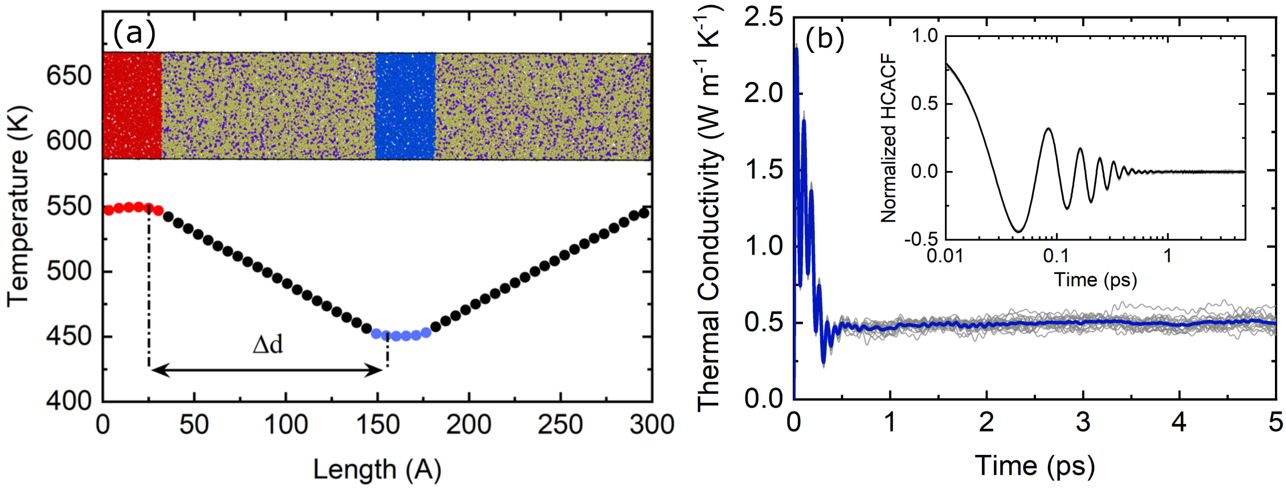

Supplementary Figure 23. Thermal conductivity for a-Si_{20}-heavySi_{80} from (a) non-equilibrium and (b) equilibrium molecular dynamic calculations. Both method result in thermal conductivity of ~0.50 W m^{-1} K^{-1}. The heavySi mass in these simulations is similar to that of the Te.

Supplementary Note 4

Minimum limit thermal conductivity. A lower limit to the thermal conductivity of materials is estimated using the minimum limit model derived from the kinetic theory of gases that works on the basis of propagating modes or phonons [19]:

$$\kappa_{\mathrm{min,\mathbf{P}}} = 1.21 k_B n^{2/3} v_g, \tag{1}$$

where k_B is the Boltzmann constant, v_g is the average sound velocity in the material, and n is the number density. The average sound velocity can be written in terms of the longitudinal (v_L) and transverse (v_T) sound velocities as:

$$v_g = \left(\frac{1}{3}\left[\frac{2}{v_T^2} + \frac{1}{v_L^2}\right]\right)^{-1/2} \tag{2}$$

In this study, for consistency, we assume that transverse sound velocity is 60% of that of the longitudinal mode, $v_T = 0.6 v_L$. For $Si_{19}Te_{81}$, using our sound velocities derived from the picosecond acoustics measurements, we measure a longitudinal sound velocity of $v_L = 2150 \pm 100$ m s^{-1}, which is in good agreement with previously reported values ($v_L = 2030$ m s^{-1}) for the sound speed of a thicker SiTe film [20]. Other parameters that are used as an input to calculate the minimum limit such as number density and sound velocities across different compositions are given in Supplementary Note 3. Using these parameters, we determine a minimum thermal conductivity of 0.24 W m^{-1} K^{-1} for $Si_{19}Te_{81}$ at room temperature. This estimate for the minimum thermal conductivity, however, is still more than a factor of two higher that the measured thermal conductivity of $Si_{19}Te_{81}$ (0.10 W m^{-1} K^{-1}). In fact, for nearly all compositions of SiTe studied in this work, Eq. 1 overpredicts the measured thermal conductivities, as shown in Supplementary Fig. 2(c). This discrepancy increases as the SiTe coordination number decreases and the alloy transitions into an under-constrained network.

Although phonon-mediated minimum limit to thermal conductivity described above and presented in Eq. 1 has served as a successful approach to predict the thermal conductivity of a variety of disordered crystals and amorphous materials, several recent works have experimentally measured values well below this limit. This has motivated others to model the thermal conductivity in amorphous solids as a form of energy hopping between localized vibrational eigenstates. According to Allen and Feldman (AF) [21], a large portion of heat in disordered solids is transferred by quantized vibrations that are neither localized nor propagating. These delocalized non-propagating vibrational modes, *diffusons*, carry heat by diffusion with wavelength on the order of the inter-atomic spacing. Based on the AF formalism, Agne *et al.* [22] suggested a modified minimum limit model for heat transport in disordered solids that relies on the concept of diffusons rather than propagating modes. They argued that in a disordered solid, the lower bound to thermal conductivity occurs when the

thermal transport is entirely driven by diffusons. This approach, albeit with the heat transfer carrier length scale being fundamentally different from those modeled in Eq. 1, leads to a similar functional form for the thermal conductivity of disordered materials:

$$\kappa_{\mathrm{min,\mathbf{D}}} \approx 0.76 P k_B n^{2/3} v_g. \tag{3}$$

According to this diffuson-mediated minimum model, in one period of oscillation, each vibrating carrier will make two attempts to transfer energy where P is the probability of a successful energy transfer. In the high temperature limit and maximum diffusivity where $P = 1$, the calculated thermal conductivity is $\sim$37% lower than the phonon minimum limit model. By applying this diffuson-mediated minimum limit model to $Si_{19}Te_{81}$, we find a thermal conductivity of 0.14 W m^{-1} K^{-1}, which is in a better agreement with the measured value. However, considering the fact that this model is supposed to set the lower bound to thermal conductivity, it still predicts 40% higher thermal conductivity than the measured value. This implies that the thermal transport in $Si_{19}Te_{81}$ is driven by other atomistic properties that impede the transfer of energy beyond those accounted for in the diffuson limit.

To resolve this, we revisit an assumption that was made in the diffuson-mediated thermal conductivity (Eq. 3), which assumes 100% of attempts to transfer energy are successful between diffusons. As discussed earlier, since the coordination number in SiTe decreases by increasing the Te concentration, the alloy transitions from an over-constrained to an under-constrained network. This reduction in the number of bonds per atom eliminates the number of pathways through which diffusons can interact, and leads to a reduction in the probability of their successful energy transfer. To account for this probability, we assume that there is linear a relationship between the coordination number and the probability of successful energy transfer. We take the element with the highest possible coordination number, in this case Si, as the maximum probability of successful energy transfer $P = 1$. Then, we calculate P for each alloy's configuration by normalizing their coordination number with respect to pure Si, i.e., $P = (<r_{\mathrm{m}}>/<r_{\mathrm{max}}>)^{1/3} = (<r_{\mathrm{Si_xTe_{1-x}}}>/<r_{\mathrm{Si}}>)^{1/3}$. Using this assumption, P changes from 1 to 0.8 for Si with $< r >$ = 4 and Te with $< r >$ = 2. By applying this condition, we calculate the diffuson-mediated thermal conductivity for $Si_{20}Te_{80}$ as 0.12 W m^{-1} K^{-1}, in better agreement with the measured values of our SiTe alloys across the compositional phase space, shown in Fig. 2(c) in the main manuscript.

Supplementary Table 3. The longitudinal v_{long}, total sound speed v_g, number density n, coordination number $< r >$, and probability of successful transfer P for different amorphous chalcogenide compositions used in thermal conductivity estimation.

Composition	v_{long} (m s^{-1})	v_g (m s^{-1})	$n \times 10^{28}$ (m^{-3})	$< r >$	P	$\kappa_{\min,\mathbf{P}}$ (W m^{-1} K^{-1})	$\kappa_{\min,\mathbf{D}}$ (W m^{-1} K^{-1})	$\kappa_{\exp}$ (W m^{-1} K^{-1})
Se	1840 [23]	1221	3.68	2.00	0.79	0.22	0.11	0.12
Te	1840 [a]	1238	2.50	2.00	0.79	0.20	0.10	0.23 [b]
$Si_{11}Te_{89}$	1980	1333	3.00	2.22	0.82	0.21	0.11	0.10
$Si_{19}Te_{81}$	2150	1447	3.05	2.38	0.84	0.24	0.12	0.10
$Si_{30}Te_{70}$	1968	1324	3.12	2.60	0.87	0.22	0.11	0.10
$Si_{34}Te_{66}$	3290	2215	3.19	2.68	0.88	0.35	0.19	0.18
$Si_{38}Te_{62}$	3153	2122	3.15	2.76	0.88	0.37	0.21	0.20
$Si_{53}Te_{47}$	3523	2372	3.35	3.06	0.91	0.41	0.24	0.30
$Si_{56}Te_{44}$	3874	2608	3.39	3.12	0.92	0.45	0.26	0.32
Si	6310 [15]	4248	4.99	4.00	1.00	0.96	0.61	0.94

[a] We assume amorphous tellurium sound speed is similar to that of selenium [23].
[b] Due to low T_g in tellurium film, it turned to crystalline during the deposition process.

Supplementary Note 5

Diffusivity and thermal conductivity calculations. In order to determine the contribution of non-propagating delocalized diffusons in thermal transport, it is necessary to calculate the thermal conductivity due to diffuson contribution. For this, we use formalism proposed by Allen-Feldman as follows:

$$\kappa_{AF} = \frac{1}{V}\sum_{i=1}^{N} C(\omega_i)D(\omega_i), \tag{4}$$

where V is the system volume, i is the mode number, N is the total number of modes, ω_i is frequency of ith mode, $C(\omega_i)$ and $D(\omega_i)$ are the frequency dependant specific heat and mode diffusivity, respectively. These parameters can be obtained by:

$$\mathbf{C}(\omega_i) = k_B[\frac{\hbar\omega_i/2k_BT}{\sinh(\hbar\omega_i/2k_BT)}]^2, \tag{5}$$

$$\mathbf{D}(\omega_i) = \frac{\pi V^2}{3\hbar^2\omega_i^2}\sum_{j}^{\neq i}|S_{ij}|^2\delta(\omega_i - \omega_j), \tag{6}$$

Where S_{ij} is the heat current operator with unit of J/m^2/s which can be obtained with the knowledge of eigenvectors, dynamical matrix, and the minimum distance between the pairs:

$$\mathbf{S}_{ij} = \frac{\hbar}{2V}\mathbf{v}_{\mathbf{K}ij}(\omega_{\mathbf{K}i} + \omega_{\mathbf{K}j}), \tag{7}$$

$$\mathbf{v}_{\mathbf{K}ij} = \frac{i}{2\sqrt{\omega_{\mathbf{K}i}\omega_{\mathbf{K}j}}} \sum_{\alpha,\beta} \sum_{m,\kappa,\kappa'} e_\alpha(\kappa;\mathbf{K},i) D^{\kappa'\kappa}_{\beta\alpha}(0,m) \times (\mathbf{R}_m + \mathbf{R}_{\kappa\kappa'}) e^{iK.\mathbf{R}_m} \times e_\beta(\kappa;\mathbf{K},i). \tag{8}$$

To obtain the AF thermal conductivity we developed our own script using MATLAB to perform the above calculations. For this, we imported the dynamical matrix, eigenvector, position, and frequency of the modes from the force constant calculations into our model and estimated the diffusivity and thermal conductivity. It is worthwhile mentioning that there is a program, General Utility Lattice Program (GULP), which automatically performs the AF thermal conductivity calculations for a limited number of potentials such as harmonic, Lennard-Jones, and Stillinger-Weber (SW) to name a few. However, since we did not use any of the existing potentials for calculating the thermal conductivity of a-$Si_{20}Te_{80}$, we had to write our own script. Nonetheless, we used GULP to validate our script by calculating the diffusivity and AF thermal conductivity of a-Si and a-SiO_2 and comparing the results with those obtained by GULP. For this, we used SW for a-Si and Beest-Kramer–van Santen (BKS) for a-SiO_2. The BKS potential is similar to those used for a-SiO_2 in previous studies [24–26]. In order to create a structure that is closer to a-$SiTe_2$, we replace the mass of oxygen to that of tellurium in a-SiO_2 system (a-$Si^{127}O_2$).

To calculate the diffusivity in a-Si, we use an amorphous system proposed by Barkema and Mousseau [27] with 1000 atoms at 300 K and under zero pressure. For a-$Si^{127}O_2$, we used melt-quench process with 720 atoms to obtain a uniform amorphous structure. The calculation parameters such as melt temperature, broadening factor, and cut-off frequency are similar to those of Ref [24–26]. To calculate the diffusivities in a-$Si_{20}Te_{80}$ a Lorentzian broadening of $1\delta\omega_{ave} = 0.4360$ cm^{-1} and a cutoff frequency if 1 cm^{-1} was used for thermal conductivity calculation. Allen and Feldman recommended a broadening factor larger than the average frequency spacing $\delta\omega_{ave}$. Considering this, we observe changing the broadening factor from $1\delta\omega_{ave} = 0.4360$ cm^{-1} to $5\delta\omega_{ave} = 2.1801$ cm^{-1} leads to small increase in the estimated thermal conductivity ($\sim$3%).

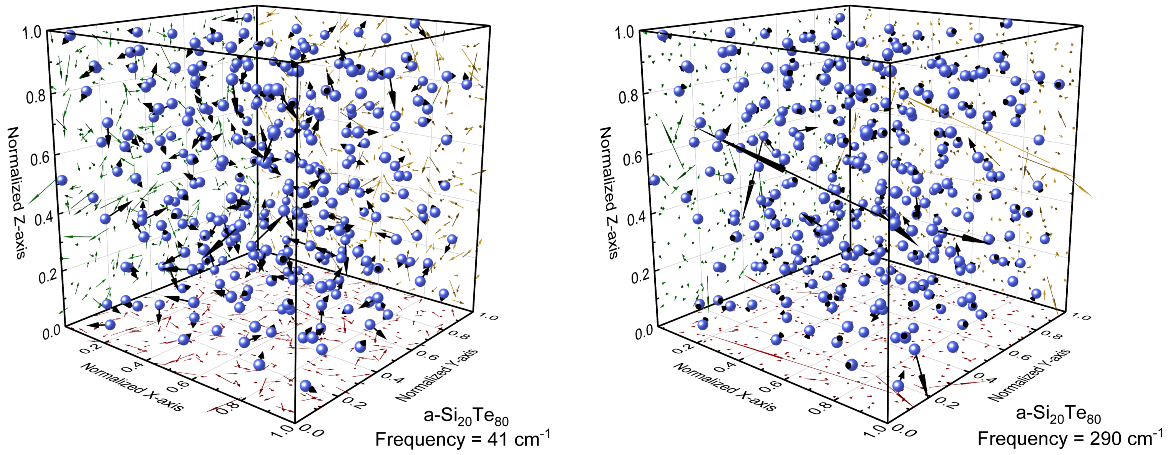

Supplementary Figure 24. (a,b) 3D Visualization of delocalized (41 cm^{-1}) and localized (290 cm^{-1}) vibrational modes on either side of the mobility edge in a-$Si_{20}Te_{80}$. The few high amplitude eigenvectors in localized frequency are an indication of strong localization showing the energy associated with these modes are confined in a small geometric region. The colored arrows represent the projection of 3D vectors into their components in the xy, yz, and xz planes.

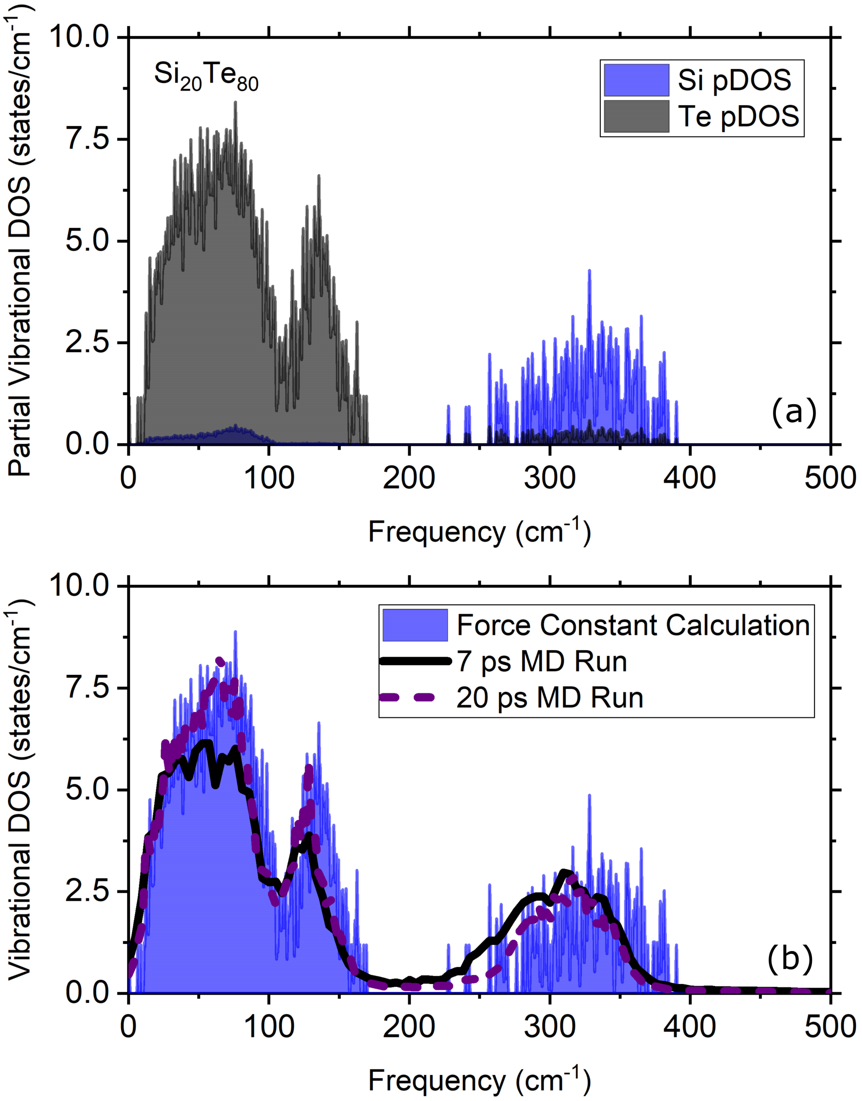

Supplementary Figure 25. (a) Partial vibrational density of states (DOS) for a-$Si_{20}Te_{80}$. (b) DOS results from force constant calculations and MD simulations with different simulation times, 7 and 20 ps.

Amorphous a-$Ge_{20}Te_{80}$ vs. a-$^{78}Si_{20}Te_{80}$. GeTe alloy is a well-known phase-change/thermoelectric material which has been extensively studied in terms of its thermal properties. Similar to silicon, germanium is a 4-coordinated element and forms a short range ordered tetrahedron upon mixing with Te. Atomic structure of SiTe studied here in many cases such as coordination number and radial distribution function resembles that of the GeTe. Supplementary Figure 26 shows the radial distribution function for our a-$^{78}Si_{20}Te_{80}$ where the mass of silicon atoms are replaced by that of germanium and a-$Ge_{20}Te_{80}$ from Ref. [28]. Due to similarities between GeTe and SiTe, it is interesting to investigate how much atomic masses of the constituent elements in a-$Si_{20}Te_{80}$ would change the thermal properties. For this, we repeat our simulations for a-$Si_{20}Te_{80}$ and change the silicon mass to that of germanium, a-$^{78}Si_{20}Te_{80}$. The result for this modified alloy system is presented in Supplementary Fig. 27. As can be seen, due to higher average atomic mass, the frequency of the modes have dropped from $\sim$400 cm^{-1} to $\sim$300 cm^{-1}. However, this has negligible impact on the thermal conductivity of

the a-$^{78}Si_{20}Te_{80}$. As discussed in the manuscript, this is because all the modes above $\sim$100 cm^{-1} are localized and do not contribute to heat transfer. Although the force constants in a-$^{78}Si_{20}Te_{80}$ has not been developed for a-$Ge_{20}Te_{80}$ alloy system, our estimated thermal conductivity is in good agreement with experimentally reported values that spans from 0.1 to 0.23 W m^{-1} K^{-1} [1, 10, 29, 30].

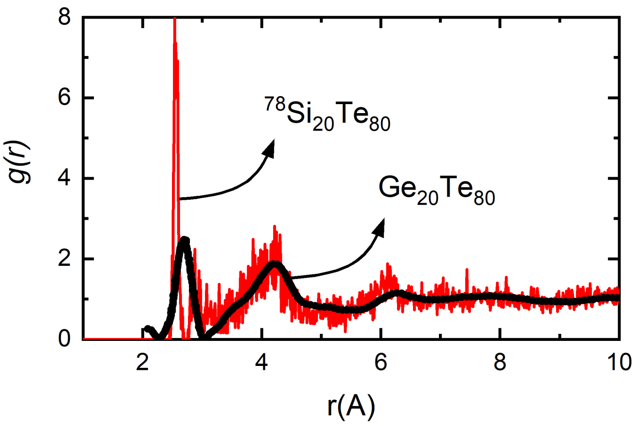

Supplementary Figure 26. Comparison between our a-$^{78}Si_{20}Te_{80}$ with 300 atom supercell and a-$Ge_{20}Te_{80}$ Ref. [28] radial distribution function.

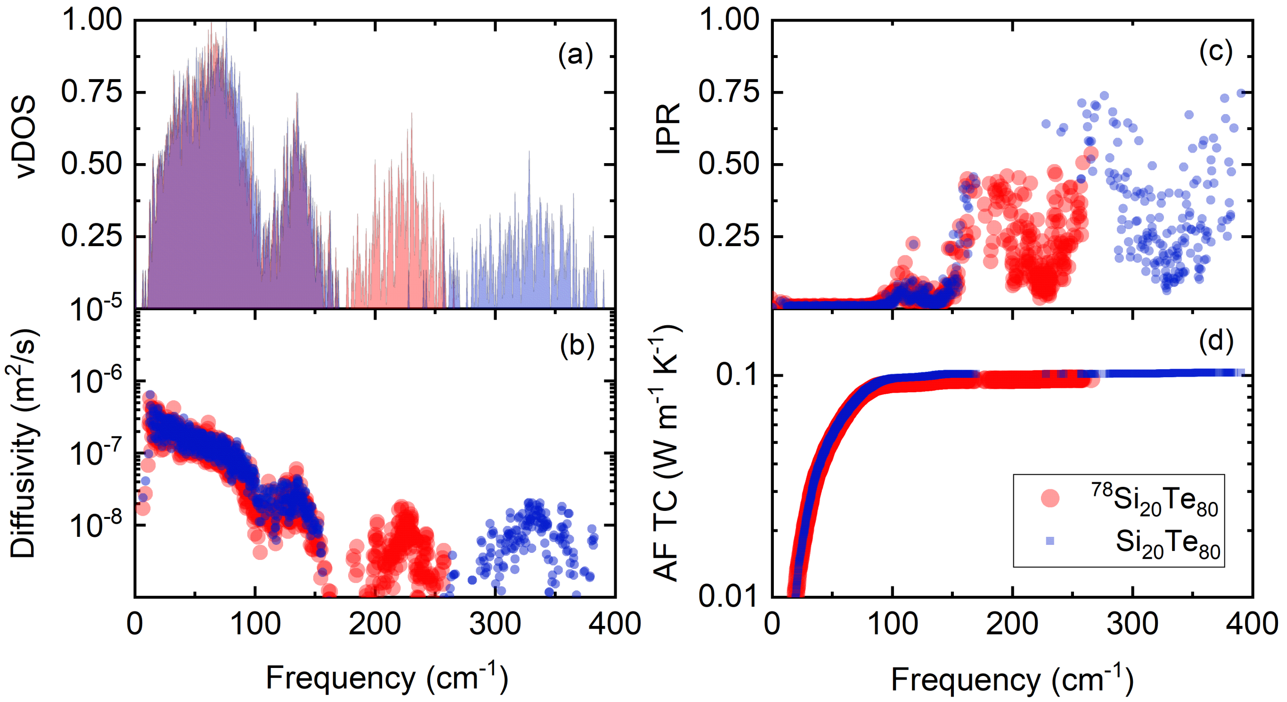

Supplementary Figure 27. (a) Vibrational density of states (vDOS) for a-$Si_{20}Te_{80}$ and a-$^{78}Si_{20}Te_{80}$ (similar mass to a-GeTe) obtained from force constant calculation on 300 atom supercell (b) diffusivity of modes calculated from AF formalism, (c) inverse participation ratio calculated from vibrational mode eigenvectors (d) Accumulative thermal conductivity as a function of modes frequency.

Supplementary Note 6

An important factor in the thermal transport mechanism is the contribution from the electrons in the total thermal conductivity. A common approach to estimate the thermal conductivity due to electron contribution is the widely used empirical equation proposed by Wiedemann-Franz (WF):

$$k = k_{\mathrm{p}} + k_{\mathrm{e}} \tag{9}$$

$$k_{\mathrm{e}} = LT/\rho \tag{10}$$

where k_p and k_e are thermal conductivities due to phonon and electron contribution, respectively, L is the Lorenz number 2.44×10^{-8} W Ω K^{-2}, T is temperature, and ρ is the electrical resistivity. According to Bailey [31] and Petersen et al. [32], the electrical resistivity of the a-Si_xTe_{1-x} for $0.02 < x < 0.4$ is in the range of 0.1-5000 Ω m. We confirmed that for the a-$Si_{20}Te_{80}$ composition the electrical resistivity in our samples is at least 100 Ω m, consistent with this range. This corresponds to electronic contribution, k_e, of 7×10^{-5}–1×10^{-9} W m^{-1} K^{-1} which are orders of magnitude lower than the measured thermal conductivity. This indicates that SiTe is electrically insulating in its room temperature unperturbed state. This is also confirmed by a more recent study, Koo et al. [33], which showed threshold switching behavior of SiTe with resistance before switching as high as ($>$ 1 GΩ at 0.1 V). The high resistivity of a-SiTe is attributed to the existence of deep trap states.

References

[1] Roberto Fallica, Enrico Varesi, Luca Fumagalli, Simona Spadoni, Massimo Longo, and Claudia Wiemer. Effect of nitrogen doping on the thermal conductivity of gete thin films. *physica status solidi (RRL)–Rapid Research Letters*, 7(12):1107–1111, 2013.

[2] E Bosoni, D Campi, D Donadio, GC Sosso, J Behler, and M Bernasconi. Atomistic simulations of thermal conductivity in gete nanowires. *Journal of Physics D: Applied Physics*, 53(5):054001, 2019.

[3] S Asokan, G Parthasarathy, and ESR Gopal. Crystallization studies on bulk sixte100- x glasses. *Journal of non-crystalline solids*, 86(1-2):48–64, 1986.

[4] Bing-Hwai Hwang. Calculation and measurement of all (002) multiple diffraction peaks from a (001) silicon wafer. *Journal of Physics D: Applied Physics*, 34(16):2469, 2001.

[5] A Wierzbicka, G Tchutchulashvili, M Sobanska, K Klosek, R Minikayev, JZ Domagala, J Borysiuk, and ZR Zytkiewicz. Arrangement of gan nanowires on si (001) substrates studied by x-ray diffraction: Importance of silicon nitride interlayer. *Applied Surface Science*, 425:1014–1019, 2017.

[6] Emil V Jelenkovic and KY Tong. Thermally grown ruthenium oxide thin films. *Journal of Vacuum Science & Technology B: Microelectronics and Nanometer Structures Processing, Measurement, and Phenomena*, 22(5):2319–2325, 2004.

[7] George T Furukawa, Martin L Reilly, and John S Gallagher. Critical analysis of heat—capacity data and evaluation of thermodynamic properties of ruthenium, rhodium, palladium, iridium, and platinum from 0 to 300k. a survey of the literature data on osmium. *Journal of Physical and Chemical Reference Data*, 3(1):163–209, 1974.

[8] Catalin Chiritescu, David G Cahill, Ngoc Nguyen, David Johnson, Arun Bodapati, Pawel Keblinski, and Paul Zschack. Ultralow thermal conductivity in disordered, layered wse_2 crystals. *Science*, 315(5810):351–353, 2007.

[9] John C Duda, Patrick E Hopkins, Yang Shen, and Mool C Gupta. Exceptionally low thermal conductivities of films of the fullerene derivative pcbm. *Physical review letters*, 110(1):015902, 2013.

[10] Sheng-Nan Zhang, Jian He, Tie-Jun Zhu, Xin-Bing Zhao, and Terry M Tritt. Thermal conductivity and specific heat of bulk amorphous chalcogenides ge20te80- xsex (x= 0, 1, 2, 8). *Journal of Non-Crystalline Solids*, 355(2):79–83, 2009.

[11] Md Abu Jafar Rasel, Ashutosh Giri, David H Olson, Chaoying Ni, Patrick E Hopkins, and Joseph P Feser. Chain-length dependence of thermal conductivity in 2d alkylammonium lead iodide single crystals. *ACS Applied Materials & Interfaces*, 12(48):53705–53711, 2020.

[12] Ashutosh Giri, Alexander Z Chen, Alessandro Mattoni, Kiumars Aryana, Depei Zhang, Xiao Hu, Seung-Hun Lee, Joshua J Choi, and Patrick E Hopkins. Ultralow thermal conductivity of two-dimensional metal halide perovskites. *Nano Letters*, 2020.

[13] Li-Dong Zhao, Shih-Han Lo, Yongsheng Zhang, Hui Sun, Gangjian Tan, Ctirad Uher, Christopher Wolverton, Vinayak P Dravid, and Mercouri G Kanatzidis. Ultralow thermal conductivity and high thermoelectric figure of merit in snse crystals. *Nature*, 508(7496):373, 2014.

[14] Woochul Lee, Huashan Li, Andrew B Wong, Dandan Zhang, Minliang Lai, Yi Yu, Qiao Kong, Elbert Lin, Jeffrey J Urban, Jeffrey C Grossman, et al. Ultralow thermal conductivity in all-inorganic halide perovskites. *Proceedings of the National Academy of Sciences*, 114(33):8693–8697, 2017.

[15] IR Cox-Smith, HC Liang, and RO Dillon. Sound velocity in amorphous films of germanium and silicon. *Journal of Vacuum Science & Technology A: Vacuum, Surfaces, and Films*, 3(3):674–677, 1985.

[16] Daniel P Sellan, Eric S Landry, JE Turney, Alan JH McGaughey, and Cristina H Amon. Size effects in molecular dynamics thermal conductivity predictions. *Physical Review B*, 81(21):214305, 2010.

[17] Takuma Hori, Takuma Shiga, and Junichiro Shiomi. Phonon transport analysis of silicon germanium alloys using molecular dynamics simulations. *Journal of Applied Physics*, 113(20):203514, 2013.

[18] Ashutosh Giri, Brian F Donovan, and Patrick E Hopkins. Localization of vibrational modes leads to reduced thermal conductivity of amorphous heterostructures. *Physical Review Materials*, 2(5):056002, 2018.

[19] David G Cahill and Robert O Pohl. Lattice vibrations and heat transport in crystals and glasses. *Annual review of physical chemistry*, 39(1):93–121, 1988.

[20] L. A. Kulakova, V. Kh. Kudoyarova, B. T. Melekh, and V. I. Bakharev. Electrical, acoustic, and acoustooptical properties of si(ge)-se-te glasses. *Chalcogenide Letters*, 2:83, 2005.

[21] Philip B Allen and Joseph L Feldman. Thermal conductivity of disordered harmonic solids. *Physical Review B*, 48(17):12581, 1993.

[22] Matthias T Agne, Riley Hanus, and G Jeffrey Snyder. Minimum thermal conductivity in the context of diffuson-mediated thermal transport. *Energy & Environmental Science*, 11(3):609–616, 2018.

[23] VF Kozhevnikov, WB Payne, JK Olson, A Allen, and PC Taylor. Sound velocity in liquid and glassy selenium. *Journal of non-crystalline solids*, 353(32-40):3254–3259, 2007.

[24] Hamid Reza Seyf and Asegun Henry. A method for distinguishing between propagons, diffusions, and locons. *Journal of Applied Physics*, 120(2):025101, 2016.

[25] Jason M Larkin and Alan JH McGaughey. Thermal conductivity accumulation in amorphous silica and amorphous silicon. *Physical Review B*, 89(14):144303, 2014.

[26] AJH McGaughey and M Kaviany. Thermal conductivity decomposition and analysis using molecular dynamics simulations: Part ii. complex silica structures. *International Journal of Heat and Mass Transfer*, 47(8-9):1799–1816, 2004.

[27] GT Barkema and Normand Mousseau. Event-based relaxation of continuous disordered systems. *Physical review letters*, 77(21):4358, 1996.

[28] I Kaban, Th Halm, W Hoyer, P Jovari, and J Neuefeind. Short-range order in amorphous germanium–tellurium alloys. *Journal of non-crystalline solids*, 326:120–124, 2003.

[29] António Pereira Gonçalves, Elsa Branco Lopes, Olivier Rouleau, and Claude Godart. Conducting glasses as new potential thermoelectric materials: the cu–ge–te case. *Journal of Materials Chemistry*, 20(8):1516–1521, 2010.

[30] Ronald J Warzoha, Brian F Donovan, Nicholas T Vu, James G Champlain, Shawn Mack, and Laura B Ruppalt. Nanoscale thermal transport in amorphous and crystalline gete thin-films. *Applied Physics Letters*, 115(2):023104, 2019.

[31] LG Bailey. Preparation and properties of silicon telluride. *Journal of Physics and Chemistry of Solids*, 27(10):1593–1598, 1966.

[32] Kurt E Petersen, Ulrich Birkholz, and David Adler. Properties of crystalline and amorphous silicon telluride. *Physical Review B*, 8(4):1453, 1973.

[33] Yunmo Koo, Sangmin Lee, Seonggeon Park, Minkyu Yang, and Hyunsang Hwang. Simple binary ovonic threshold switching material site and its excellent selector performance for high-density memory array application. *IEEE Electron Device Letters*, 38(5):568–571, 2017.
